# Supplementary material for: The maximal and current accuracy of rigorous protein-ligand binding free energy calculations
Source: Commun Chem. 2023 Oct 14;6:222. doi: 10.1038/s42004-023-01019-9 (PMC10576784; doi:10.1038/s42004-023-01019-9)
Supplement: Supplementary file 2 — Supplementary Information [file 42004_2023_1019_MOESM2_ESM.pdf]

SUPPLEMENTARY INFORMATION FOR THE MAXIMAL AND CURRENT ACCURACY OF RIGOROUS PROTEIN-LIGAND BINDING FREE ENERGY CALCULATIONS

Gregory A. Ross[*†]
Schrödinger Inc,
New York, NY, USA
gregoryaross@isomorphiclabs.com

Chao Lu
Schrödinger Inc,
New York, NY, USA

Guido Scarabelli
Schrödinger Inc,
New York, NY, USA

Steven K. Albanese
Schrödinger Inc,
New York, NY, USA

Evelyne Houang
Schrödinger Inc,
New York, NY, USA

Robert Abel
Schrödinger Inc,
New York, NY, USA

Edward D. Harder
Schrödinger Inc,
New York, NY, USA

Lingle Wang[†]
Schrödinger Inc,
New York, NY, USA
lingle.wang@schrodinger.com

September 11, 2023

[*]Present address: Isomorphic Labs, London, UK
[†]These authors jointly supervised this work

Contents

1 Supplementary Methods **3**
1.1 Experimental reproducibility survey data . . . 3
1.1.1 Experimental uncertainty from repeating the same assay . . . 5
1.2 The expanded conformer approach . . . 5
1.2.1 Correcting under-sampled symmetric rotamers . . . 6

2 Supplementary Results **6**
2.1 FEP+ benchmark system development . . . 6
2.1.1 The public Merck data sets . . . 6
2.1.2 FEP+ fragment data set . . . 15
2.1.3 GPCRs . . . 16
2.1.4 Janssen BACE1 data sets . . . 20
2.1.5 Bayer macrocycles . . . 24
2.1.6 MCS docking sets . . . 25

2.1.7 FEP+ scaffold-hopping 26
2.1.8 FEP+ macrocycles 26
2.1.9 FEP+ buried water set 28
2.1.10 FEP+ charge change set 28
2.1.11 Miscellaneous data sets 28
2.1.12 FEP+ R-group set 30
2.1.13 OPLS stress set 33
2.1.14 OPLS drug discovery 34
2.2 On the variance of accuracy from different FEP+ approaches 34

1 Supplementary Methods

1.1 Experimental reproducibility survey data

Supplementary Tables 1, 2, and 3 summarize the data that was used in the experimental reproducibility survey. An attempt has been made to separate the comparisons by type, namely comparisons of different binding assays (Supplementary Table 1), comparisons of binding assays and inhibition assays (Supplementary Table 2), and comparisons of two different inhibition assays (Supplementary Table 3).

In most cases, the affinity and inhibition data was taken directly from tables in the original studies. In two cases [1;2], the experimental binding data was extracted by digitizing scatter plots. In the process of collecting these data, other assay comparison types were encountered, such as cases were the exactly the same assay was repeated by different experimentalists [3–5], cases where the same assay was performed with a different buffer or membrane model [6;7], and a case where an assay was repeated with a different type of NMR label [8]. These cases and others like them were not included in this study as the assays in the comparisons were deemed to be too similar.

Supplementary Table 1: A summary of the data that was used to evaluate the reproducibility of binding assays against different binding assays. Each row represents a comparison of two types of assay; the 'No. compounds' column refers to the number of identical compounds that were used in both assays. The mean unsigned error (MUE) and root-mean-square error (RMSE) have been calculated using all pairs of relative binding affinities within each study. The number of relative binding affinities in each assay comparison are listed in the 'No. pairs' column. Regarding the source of the data, the first author and year of each study is shown in the first column, along with the tables and/or figures from which the affinity values were taken from.

Study and source of data	Protein and assay descriptions	No. compounds	No. pairs	Pairwise MUE (kcal mol^{-1})	Pairwise RMSE (kcal mol^{-1})
Mandine 2001 [9], tables 1 and 2	SH2: SPA vs SPR	17	136	1.2	1.54
Murphy 2006 [10], table 1	Herg: Binding assays	5	10	1.19	1.35
Navratilova 2007 [11], table 2	Ca II: SPR vs ITC	4	6	0.13	0.14
Murthy 2008 [12], tables 1 and 2	Lectin: SPR vs ITC	6	15	0.4	0.45
Hang 2009 [1], tables 1 and S1	HCV polymerase (Con) SPR vs fluorescence	4	6	0.79	0.95
Jecklin 2009 [6], table 1	Ca I: nESI-MS vs ITC	5	10	0.44	0.5
	Ca I: SPR vs ITC	7	21	1.48	1.83
	Ca I: nESI-MS vs SPR	6	15	2.18	2.63
Aaron 2010 [13], tables 2.1 and 2.2	Ca II: SPR vs ITC	3	3	1.29	1.4
Mason 2012 [14], table 1	FAK: SPR vs LanthaScreen	5	10	0.79	0.95
Newman 2012 [15], table 1	Trypsin: SPR vs ITC	7	21	0.33	0.39
Rogez-Florent 2014 [16], table 1	Ca II (h & b) SPR vs ITC	8	12	0.22	0.26
	Ca II (h & b) SPR vs TSA	8	12	0.71	0.82
	Ca II (h & b) ITC vs TSA	8	12	0.81	0.95
Schnapp 2016 [17], figures 2 and 3	DPPIV: SPR vs ITC	9	36	1.62	1.94
Ycas et al. 2020 [8], tables 1 and 3	BPTF: NMR vs SPR	4	6	0.98	1.1
	BPTF: NMR vs SPR labelled	4	6	1.14	1.28
	BPTF: NMR vs alpha screen	4	6	0.77	0.85
	BPTF: SPR vs alpha screen	7	21	0.39	0.45
	BPTF: Labeled SPR vs alpha screen	7	21	0.42	0.49
Perterson 2018 [18], tables 6 and 7	Galectin-3: FP vs ITC	10	45	0.37	0.47

Supplementary Table 2: A summary of the data that was used to compare binding assay affinity data with functional inhibition assays. Two data points from Patil 2019 were removed from our analysis as they were clearly beyond the functional assay detection limit. A description of the columns can be found in the caption of Supplementary Table 1.

Study and source of data	Protein and assay description	No. compounds	No. pairs	Pairwise MUE (kcal mol^{-1})	Pairwise RMSE (kcal mol^{-1})
Nikolovska-Coleska 2004 [19], table 3	XIAP BIR3: Ki vs Kd	6	15	0.49	0.65
Baum 2009 [20], figure 1 and table 1	Thrombin: Ki vs Kd	12	66	0.63	0.72
Hang 2009 [1], figures A and B	HCV polymerase (Con): IC50s vs Kd	33	528	1.05	1.34
	HCV polymerase (BK): IC50s vs Kd	32	496	0.99	1.25
Kung 2011 [21], abstract and table 1	HSP90: Functional competition SPA vs ITC	4	6	0.62	0.72
Mason 2012 [14], table 1	FAK: Inhibition vs SPR	10	45	0.78	0.92
	FAK: Inhibition vs LanthaScreen	5	10	1.15	1.51
Li 2016 [22], tables 2 and 4	DPPIV: functional inhibition vs SPR	4	6	0.93	1.07
Schnapp 2016 [17], figure 2 and table S1	DPPIV: functional inhibition vs SPR	9	36	1.01	1.2
	DPPIV: functional inhibition vs ITC	9	36	0.92	1.09
Crawford 2016 [23], table S4	BRD4(1): BROMScan (KD) vs TR-FRET (IC50)	4	6	0.23	0.25
	BRD4(2): BROMScan (KD) vs TF-FRET (IC50)	4	6	0.29	0.33
	BRD9: BROMScan (KD) vs TF-FRET (IC50)	4	6	0.36	0.43
	BRPF1: BROMScan (KD) vs TF-FRET (IC50)	4	6	0.24	0.28
	CECR2: BROMScan (KD) vs TF-FRET (IC50)	4	6	0.29	0.32
	TAF1(2): BROMScan (KD) vs TF-FRET (IC50)	4	6	0.32	0.42
	CECR2: BROMScan (KD) vs TF-FRET (IC50)	4	6	0.23	0.28
Patil 2019 [24], tables 1 and 2 (column 'Affinity')	AChE: enzyme activity vs SPR affinity	9	36	1.32	1.62
Schindler et al. 2020 [2], figure S2	SPR vs functional assay	8	28	1.4	1.89

Supplementary Table 3: A summary of the data that was used to compare functional inhibition assays with other functional inhibition assays. Measurements that were clearly at the top and bottom of the assays in the Jia 2006 and Covid Moonshot studies were removed from this analysis. A description of the columns can be found in the caption of Supplementary Table 1.

Study and source of data	Protein and assay description	No. compounds	No. pairs	Pairwise MUE (kcal mol^{-1})	Pairwise RMSE (kcal mol^{-1})
Jia 2006 [25], digitized from figure 5b	COT kinase: IC50s vs IC50s	53	1378	0.41	0.50
Katz 2001 [26] table 1, Katz 2003 [27] table 1	uPA: Kis vs Kis (ligands 8696, 10302, 11092)	3	3	0.73	0.78
Chen 2013 [28], table 3	Angiotensin I-converting enzyme: IC50s vs IC50s	3	3	0.04	0.05
Covid Moonshot 2020 activity data [29],	SARS-CoV-2 main protease: Kis vs Kis	324	52326	0.60	0.79

Supplementary Table 4: Comparative assays of five targets from our internal drug discovery projects. These data serve as a validation of the publicly available comparative data. The averages are weighted by the number of ligands in each assay comparison and square brackets encompass 95% confidence intervals that have been calculated by bootstrap sampling over the projects.

Project	Assay types	No. compounds.	Pairwise MUE	Pairwise RMSE	R^2	Kendall τ
A	Binding vs functional	706	0.57	0.77	0.83	0.75
B	Binding vs functional	22	1.34	1.71	0.70	0.70
C	Binding vs functional	27	0.85	1.03	0.52	0.42
D	Binding vs binding	56	0.57	0.73	0.89	0.77
	Binding vs functional	12	0.88	1.1	0.8	0.64
	Binding vs functional	42	0.84	1.16	0.68	0.67
	Binding vs functional	22	0.68	0.84	0.87	0.6
	Binding vs functional	52	0.92	1.19	0.69	0.63
	Functional vs functional	13	0.66	0.84	0.89	0.67
E	Binding vs binding	56	0.77	0.99	0.79	0.69
	Binding vs functional	12	0.86	1.05	0.77	0.64
	Binding vs functional	53	0.48	0.62	0.91	0.84
	Binding vs functional	24	0.85	1.04	0.75	0.53
	Binding vs functional	66	0.88	1.14	0.72	0.63
	Functional vs functional	14	0.59	0.73	0.91	0.74
	Total/average	1177	0.65 [0.59, 0.86]	0.88 [0.80, 1.13]	0.81 [0.73, 0.83]	0.72 [0.71, 0.75]

1.1.1 Experimental uncertainty from repeating the same assay

In this section, we collect different estimates of the uncertainty that comes from repeating the same affinity assay more than once. Standard deviations from measurements that were reported as Kds, Kis, or IC50s were converted into kcal mol^{-1} at 300 K. If σ_{K_d} is the standard deviation of the binding affinity K_d and μ_{K_d} is the mean of K_d, the standard deviation of the binding free energy, denoted $\sigma_{\Delta G}$, can estimated as

$$\sigma_{\Delta G} = kT \frac{\sigma_{K_d}}{\mu_{K_d}}, \qquad \text{(Supplementary Equation 1)}$$

which comes from the fact that $\left| \frac{d(\Delta G)}{dK_d} \right| = \frac{kT}{K_d}$.

Supplementary Table 5: Root-mean-square (RMS) of the standard deviations of repeated measurements of protein-ligand binding affinities (in kcal mol^{-1}). The overall RMS on the last row is the square-root of the weighted mean-square standard deviation from each study, where each study was weighted by the number of compounds. The academic data sources are as follows: CA II binding assays was taken from table 3 (participants 10 and 14) from Myszka et al.[3], data from the Herg binding assay was taken from figure 5 from Murphy et al.[10], the SPR data for DPPIV was taken from figures 2 and 5 from Schnapp et al.[17], the DPPIV ITC data was taken from figures 3 and 6 from Schnapp et al.[17], the galecting FP data was taken from tables 1 to 7 from Perterson[18], the galectin ITC data was taken from tables 6 and 7 from Perterson[18], and the MCL1 data was taken from table 3 from Friberg et al.[30]. Projects B, C, and D from Schrödinger's internal drug discovery projects. All values in the rightmost column are standard deviations, with the possible exception of the galectin and MCL1 data as it is unclear from the sources whether the reported values are standard deviations or standard errors.

Study and assay type	No. compounds	No. repeats per compound	RMS standard deviation (kcal mol^{-1})
CA II, binding (ITC)[3]	1	Between 3 and 5	0.20
Herg binding[10]	8	2	0.28
DPPIV, binding (SPR)[17]	9	2	0.11
DPPIV, binding (ITC)[17]	9	At least 2	0.32
Galectin3, binding (FP)[18]	33	At least 2	0.058
Galectin3, binding (ITC)[18]	10	Unknown	0.70
MCL1, functional[30]	42	3	0.15
Project B, functional	22	Between 2 and 104	0.26
Project B, binding 1	8	Between 2 and 6	0.37
Project B, binding 2	6	Between 2 and 5	0.43
Project C, functional	16	Between 2 and 76	0.19
Project D, functional 1	31	Between 2 and 8	0.21
Project D, binding	98	Between 2 and 24	0.17
Project D, functional 2	9	Between 2 and 3	0.38
Project D, functional 3	48	Between 2 and 10	0.16
Totals	350		0.23 [0.18, 0.33]

1.2 The expanded conformer approach

Before running FEP, users have to decide on the binding geometry of the ligands. Typically, users opt for a one single structure per ligand that has been predicted by docking or by similarity to a crystal structure. If there are multiple possible poses to choose from, one could use FEP to select which pose to use, either via a relative binding free energy protocol[31] or absolute binding free energy protocol[32]. Selecting the binding geometry using free energy calculations benefits from the increased sampling and accuracy afforded by all-atom simulations. A significant drawback to selecting binding geometries in this way is that they require restraints that maintain the geometries somewhat fixed during the calculations. The use of restraints can be cumbersome and requires the application of corrections in the calculated free energies.

For ligands that have more than one possible binding conformation, we sought an approach to avoid using restraints that could easily be incorporated into the FEP+ workflow. We restricted our attention to cases where the binding site of

the protein naturally restricts the conformation of the ligand in the complex simulations but where the ligand can freely sample conformations in the solvent simulations. Here, we treat additional binding modes as extra ligands within the FEP+ workflow. This way, as well as outputting the calculated binding free energies between the ligands, FEP+ will also calculate the relative binding free energies between different binding modes. Rather than selecting a single pose to use, we apply a statistical mechanical correction, outlined below, that handles all binding conformations at once.

Consider an edge that connects 2 different binding conformations of the same ligand. If, in the solvent simulations, the ligand adopts a distribution of conformations that is independent of the starting conformation, then the calculated solvent free energy difference would be zero. If, in the complex simulations, the ligand cannot adopt the alternate conformation when the simulation was started in the other, then the binding free energy difference is equal to the relative binding free energy of the two confirmations.

Without additional processing, FEP+ will treat each binding conformation as having a different predicted binding free energy and so a correction is required to account for the additional binding conformations. With the cycle closure correction algorithm, FEP+ produces absolute binding free energies up to an unknown additive constant. Let ΔG_i denote this absolute binding free energy for a particular ligand in binding conformation i and let there be a total of n binding conformations for that ligand. If ΔG denotes the absolute binding free energy for the ligand that fully accounts for all the binding modes, then ΔG_i and ΔG are related to one another by the following equation:

$$\Delta G = \Delta G_i - kT \ln \left(1 + \sum_{i \neq j}^{n} e^{-\Delta\Delta G_{ij}/kT} \right), \qquad \text{(Supplementary Equation 2)}$$

where $\Delta\Delta G_{ij}$ is the relative binding energy between conformations i and j. This equation is valid as long as the binding conformations do not interconvert in the complex simulations but can readily interconvert in the solvent simulations. The use of Supplementary Equation 1 is only approximate if the solvent simulations do not produce conformational ensembles that are independent of the starting conformation. Additionally, the estimate ΔG will be erroneously biased to more negative free energies if the conformations in the complex can interconvert.

1.2.1 Correcting under-sampled symmetric rotamers

Supplementary Equation 2 is general and can be applied to multiple conformations of any type. One type of conformational degree of freedom are R-groups that have rotational torsion symmetry. A common example is a terminal phenyl group, which has a two-fold rotational symmetry. An estimate of the binding free energy that is too positive will occur if an R-group with rotational symmetry samples its symmetric rotamer torsions freely and equally in solvent but is stuck in its starting torsion angle in the protein. As the $\Delta\Delta G_{ij}$ between two symmetric rotamer states i and j is zero, Supplementary Equation 2 tells us that we have underpredicted the binding free energy by $kT\ln(2)$. Thus, cases like this need to have their binding free energy lowered by $kT\ln(2)$.

2 Supplementary Results

2.1 FEP+ benchmark system development

Here, we detail our curation of the protein ligand data sets that comprise the FEP benchmark. The protein and ligand data is divided into sections that generally reflects the origin of the protein and ligand structures.

In the group summary tables, we summarize accuracy FEP using both the edgewise and pairwise root-mean-square error (RMSE) as well as the coefficient of determination (R^2) of the predicted absolute binding free energy predictions, which can be calculated up to an unknown additive constant. The mean of the pairwise RMSE and R^2 in each section are calculated as a weighted mean over FEP maps, where the weight is given by the number of compounds in the map. The edgewise RMSE is calculated by weighted each map by the number of edges. Throughout, square brackets are used to show 95% confidence which have been calculated by bootstrap sampling over the maps in each section. Supplementary Table 6 shows the aggregate statistics for each group; the statistics for each system can be found in each section below.

2.1.1 The public Merck data sets

This section outlines our development of the large publicly accessible data set that were assembled by Schindler et al.[2]. Our retrospective validation of the FEP systems includes improving the protein structures and ligand binding modes, adding additional protonation states of some ligands, as well as removing some ligands. After extending their simulations to 20ns per lambda window, Schindler reported a weighted pairwise RMSE of 1.51 [1.44, 1.73] kcal mol^{-1}. After the various model adjustments that we detail below, we report a modest reduction of the weighted pairwise RMSE to 1.34 [1.18, 1.49] kcal mol^{-1}.

Supplementary Table 6: Data sets in FEP+ benchmark have been group according to their origin or first use as an FEP data set. The accuracy for each individual statistics for system can be found in each section. This table shows the aggregate statistics for each group. The weighted means have been calculated by weighting edgewise RMSEs by the number of edges in the graph and all other statistics by the number of compounds. Confidence intervals have been calculated by bootstrap sampling over the different graphs

Group	No. compounds	No. edges	R^2	Edgewise RMSE	Pairwise RMSE
Public Merck	264	576	0.53 [0.44, 0.62]	1.15 [0.97, 1.33]	1.34 [1.17, 1.51]
FEP+ fragments	79	114	0.59 [0.43, 0.77]	0.92 [0.76, 1.06]	1.01 [0.81, 1.2]
GPCRs	98	207	0.41 [0.33, 0.5]	1.40 [1.22, 1.43]	1.55 [1.41, 1.62]
Janssen BACE1	74	127	0.50 [0.27, 0.62]	1.08 [0.83, 1.24]	1.22 [0.9, 1.45]
Bayer macrocycles	8	11	0.43 [0.15, 0.9]	0.75 [0.64, 1.0]	0.82 [0.7, 1.0]
MCS docking	49	77	0.57 [0.46, 0.74]	1.65 [1.37, 1.8]	1.46 [1.39, 1.5]
FEP+ scaffold-hopping	17	20	0.73 [0.4, 0.98]	0.78 [0.42, 1.05]	0.77 [0.47, 1.06]
FEP+ macrocycles	34	44	0.66 [0.42, 0.92]	1.09 [0.7, 1.36]	1.32 [0.78, 1.74]
FEP+ buried water	76	103	0.62 [0.45, 0.77]	1.29 [0.94, 1.61]	1.28 [0.98, 1.54]
FEP+ charge-change	53	120	0.67 [0.47, 0.84]	1.20 [1.0, 1.36]	1.20 [1.0, 1.37]
Miscellaneous	79	120	0.59 [0.26, 0.75]	0.96 [0.77, 1.18]	1.01 [0.77, 1.25]
FEP+ R-group	199	383	0.57 [0.47, 0.71]	0.98 [0.79, 1.1]	1.05 [0.88, 1.16]
OPLS stress set	114	158	0.52 [0.36, 0.69]	1.44 [1.09, 1.66]	1.44 [1.15, 1.62]
OPLS drug discovery	93	122	0.60 [0.47, 0.79]	1.03 [0.85, 1.16]	1.16 [0.91, 1.38]
Total/weighted mean	1237	2182	0.56 [0.51, 0.60]	1.17 [1.08, 1.25]	1.25 [1.17, 1.33]

Supplementary Table 7: The performance of FEP+ on the protein-ligand data sets that were initially prepared by Schindler et al. The weighted means have been calculated by weighting edgewise RMSEs by the number of edges in the graph and all other statistics by the number of compounds. Confidence intervals have been calculated by bootstrap sampling over the different graphs.

System	PDB	No. compounds	No. edges	R^2	Edgewise RMSE	Pairwise RMSE
CDK8	5CEI	32	54	0.56	1.62 ± 0.14	1.63 ± 0.05
c-Met	4R1Y	24	40	0.82	0.85 ± 0.09	1.07 ± 0.04
Eg5	3L9H	28	51	0.35	1.33 ± 0.16	1.25 ± 0.05
HIF-2α	5YBM	41	103	0.61	1.05 ± 0.07	1.10 ± 0.02
PFKFB3	6HVI	40	136	0.58	1.27 ± 0.08	1.42 ± 0.03
SHP-2	5EHR	26	39	0.50	1.31 ± 0.22	1.60 ± 0.06
SYK	4PUZ	46	101	0.51	0.80 ± 0.06	1.05 ± 0.02
TNKS2	4UI5	27	52	0.29	0.91 ± 0.14	1.61 ± 0.06
	Total/weighted mean	264	576	0.53 [0.44, 0.62]	1.15 [0.97, 1.33]	1.34 [1.17, 1.51]

CDK8

The same protein crystal structure as used by Schindler et al. (PDB code 5HNB) was re-prepared using Schrödinger's protein preparation wizard. To aid the stability of the protein structure, the cyclin that was bound to CDK8 in the crystal structure was retained in the preparation and the FEP calculations. Like the majority of CDK8 crystal structures, 5HNB has a relatively low resolution (2.35 Å) and has unresolved loops between residues between ALA 116 and LEU 123, LYS 185 and THR 196, as well as GLU 239 and SER 244. These loops were modeled with Prime. The latter two missing loops may affect the accuracy of FEP as, although they do not directly interact with the modeled ligand binding modes, they are adjacent to the binding site and involved in the activation of CDK8. Being consistent with predictions with the MCS docking procedure, the binding modes used by Schindler et al. were used as the basis in our calculations. It was found that the binding site could easily accommodate alternative rotamer states of seven ligands (see Supplementary Figure 1 and Supplementary Table 8), which were also added to the perturbation map. After the initial calculations, it was also observed that one set of ligands (for example, see Supplementary Figure 1) sampled

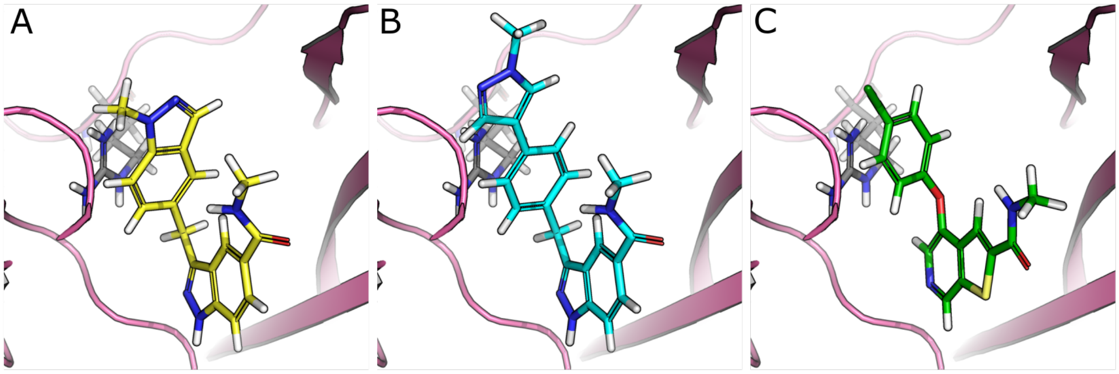

Supplementary Figure 1: Examples of ligands bound to CDK8. Panels A and B show two ligands from the Schindler et al. data set and panel C shows a ligand from the congeneric series by Koehler et al. [REF]. ARG 356 is shown in the background. Panel A shows ligand 18, which was included twice in the perturbation map to account for two rotamers of the 1-methylindazole group. The phenyl group between the indozale core and the 1-methylpyrazole group could sample the symmetrical rotations in solvent but not in the complex, meriting a symmetry correction.

the symmetrical rotamers of a phenyl group in solvent but not in the complex. Thus, for these ligands, a symmetry correction was applied.

Supplementary Table 8: The ligands in the CDK8 series from Schindler et al. that had extra rotamers added to the perturbation map.

Ligands with 2 rotamer states	13, 16, 17, 18, 42, 43, 44, 45
Ligands that required a symmetry correction	All ligands except 13, 14, 15, 16, 17, 18, 42, 43, 44, 45.

Without the extra rotamers and binding mode correction and symmetry corrections, the pairwise RMSE was 1.86 ± 0.05. After the corrections, the pairwise RMSE was reduced to 1.73 ± 0.05 kcal mol^{-1}. This high error prompted an appraisal of the accuracy of FEP+ on CDK8 in general. To aid in the investigation, a different congeneric series of 10 ligands and protein crystal structure (PDB code 5CEI) by Koehler et al.[33] were prepared as before and run with FEP+. The ligands in the set by Koehler et al. were structurally similar to the ones used by Schindler et al., but too dissimilar to reliably include in a single, unified map. On this data, FEP+ had a pairwise error of 1.34 kcal mol^{-1}, which is significantly lower than what was found with the protein and ligands from Schindler et al. The crystal structure 5CEI has a higher resolution than 5HNB (2.24 Å compared with 2.35 Å) as well as better crystal quality metrics (such as a lower R-free), although it has the same unsolved loops which require modelling by Prime. The 5HNB and 5CEI structures are very similar, with an RMSD of only 0.55 Å between the CDK8 structures after alignment. When the FEP+ calculations were repeated on the ligands from Koehler et al. with the 5HNB, the pairwise RMSE increased slightly from 1.34 ± 0.13 kcal mol^{-1} to 1.42 ± 0.13 kcal mol^{-1}. However, repeating the FEP+ calculations on the ligands from Schindler et al. with the 5CEI left the RMSE largely unchanged at 1.68 ± 0.04 kcal mol^{-1} (after the application of the corrections). Nevertheless, in the endpoints of the FEP+ calculations, the protein RMSD was found to be, on average, lower with the 5CEI structure than with 5HNB (2.05 ± 0.02 Å compared with 2.30 ± 0.03 Å). Thus, owing to the higher reliability and accuracy of the 5CEI structure, FEP results calculated with this structure were used in our overall accuracy evaluation.

Finally, inspection of the medicinal chemistry publication upon which this FEP was constructed revealed that one of the molecules (ligand 28) was at the “top”of the assay (with a measured IC_{50} of >30 μM)[34]. Removing this ligand from the final map resulted in the final pairwise RMSE of 1.62 ± 0.05 kcal mol^{-1}.

C-met

As the protein structure used by Schindler et al. lacked crystallographic water molecules, the protein-ligand structure (PDB code 4R1Y) was downloaded and prepared from scratch. Some of the ligand poses used by Schindler et al. were inconsistent with each other in the solvent facing part of the molecule. These inconsistencies unnecessarily increased the number of atoms that were considered as part of the perturbation region. Because of this, 8 ligands (listed in Supplementary Table 9) were redocked using the MCS docking protocol which resolved the inconsistencies. Supplementary Figure 2 illustrates three of the pose inconsistencies before and after redocking. The ligands CHEMBL

3402756 and CHEMBL 3402754 had an additional degree of symmetry that was sampled in the solvent but not in the complex, thus requiring a symmetry correction. Before applying the symmetry correction, the pairwise RMSE was 1.09 ± 0.04 kcal mol^{-1}. After applying the symmetry correction, the pairwise RMSE was 1.07 ± 0.04 kcal mol^{-1}.

Supplementary Table 9: The ligands in the c-met series from Schindler et al. that were redocked to resolve pose inconsistencies.

CHEMBL 3402756, CHEMBL 3402757, CHEMBL 3402758, CHEMBL 3402759, CHEMBL 3402760, CHEMBL 3402761, CHEMBL 3402762

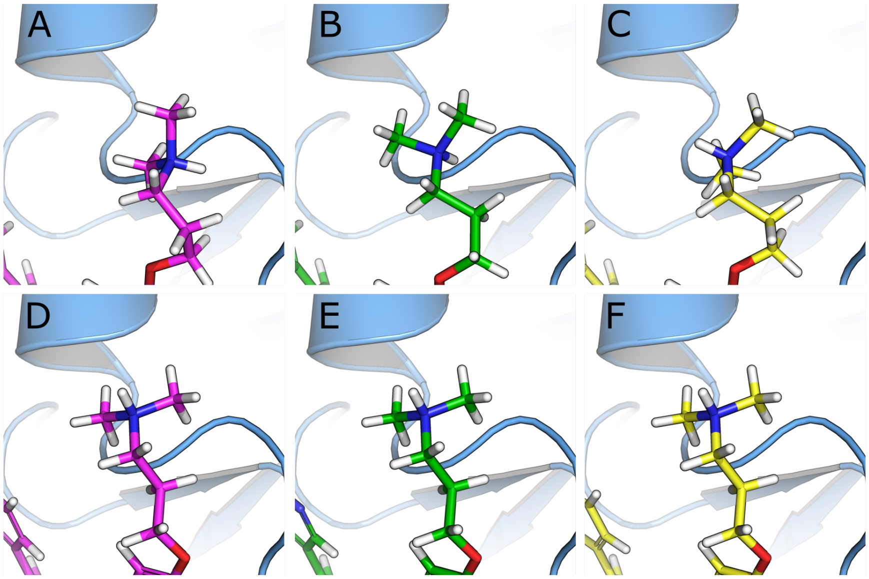

Supplementary Figure 2: Three example ligands from the C-met map that were redocked using the MCS docking protocol. The poses used by Schindler et al. - shown in panels A, B, and C - have inconsistent dimethylammonium orientations. The redocked poses - shown in panels D, E, and F - rectify this inconsistency. In the FEP+ perturbation map, the compound with magenta carbon atoms (A, D) is called CHEMBL 3402759, the compound with green carbon atoms is called CHEMBL 3402758 (B, E), and the compound with yellow carbon atoms (C, F) is called CHEMBL 3402757.

Eg5

Schindler et al. reported improved FEP accuracy after remodeling a protein loop in the vicinity of the ligands. We used their remodeled protein model for our calculations as well as the same docked ligand poses.

The series contains a number of ligands with titratable secondary and tertiary amine groups (see Supplementary Table 10). Previously, Schindler et al. treated these groups as protonated (and positively charged), which is consistent with the pH of the assay buffer (6.8)[35]. To see if the amines had a shift in the pK upon binding to Eg5, Epik was applied to predict protonation states within 2.5 pKa units of the pH of the reaction buffer. Epik produced deprotonated pairs for six ligands, whose predicted amine pKas ranged from 7.80 and 9.13. These ligands were added to the perturbation map and the Epik ionization penalty was used for the pKa correction. The addition of the protomers minimally impacted the FEP+ predictions; without the additional protamers, the pairwise RMSE was 1.27 ± 0.05 kcal mol^{-1}, whereas with the six additional protomers along with the pKa correction, the pairwise RMSE was 1.26 ± 0.05 kcal mol^{-1}. As Supplementary Table 10 shows, the pKas of the amines in the complex are all larger than the pH of the assay. Although the perturbation map with the additional deprotonated ligands hardly affected the predictions, the results from this map were used in the benchmark assessment as they remove any ambiguity regarding the protonation state of the ligands.

HIF-2α

We used the same protein structure as initially prepared by Schindler et al.. After visually inspecting the ligands and poses, we noticed that a number of ligands had a decorated phenyl ring (see Supplementary Figure 3) that could fit equally well into the binding site after rotating the ring by 180 degrees. Thus, two rotamer states were added to our perturbation map for every ligand that satisfied this criteria (listed in Supplementary Table 11). Three ligands, also

Supplementary Table 10: The pKa of the amines of six ligands in the Eg5 perturbation map. The FEP contained both the protonated and deprotonated forms of these ligands. As the pH of the assay was 6.8, the addition of the deprotonated forms minimally impacted the FEP+ predictions.

Ligand name	Solvent pKa (Epik)	Complex pKa
CHEMBL 1084143	9.06	10.21
CHEMBL 1084676	8.47	8.54
CHEMBL 1084677	9.13	10.48
CHEMBL 1084935	7.8	7.2
CHEMBL 1096002	8.64	8.79
CHEMBL 1096003	8.1	9.25

listed in Supplementary Table 11, had bi-fluorinated phenyl rings with a rotational degree symmetry equal to two. These symmetrical rotamer states could be easily sampled in the solvent, but steric barriers prevented the rotation of the symmetrical rotation in the complex. For these three ligands (listed in Supplementary Table 11), a rotation symmetric correction was applied.

Supplementary Table 11: Listing the ligands in HIF-2α that had either a rotamer or symmetry correction applied. The names of the ligands are as they appear in the perturbation map.

Ligands with 2 rotamer states	1, 25, 41, 50, 54, 61, 7a, 7b, 84, 124.
Ligands that required a symmetry correction	23, 67, 251.

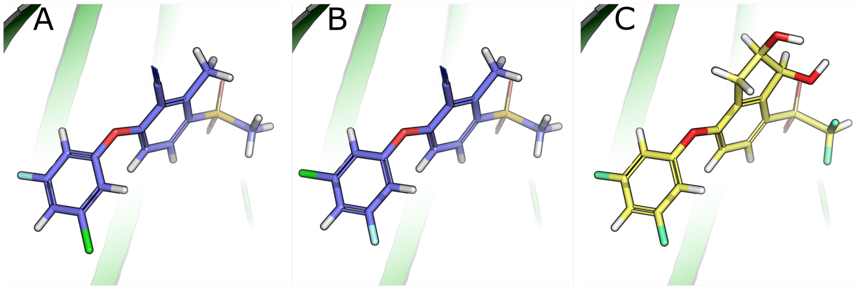

Supplementary Figure 3: Examples of HIF-2α ligands that required corrections. Panels A and B show the binding modes that were used for ligand 50. Panel A shows the two binding mode as initially prepared by Schindler et al.. The binding site can sterically accommodate the alternate rotamer state of the bi-halogenated phenyl ring. Panel B shows this alternate rotamer state that was also included in our perturbation map. These two rotamer states were processed in binding mode correction scipt. Panel C shows ligand 23, whose bi-flurpnated phenyl ring samples symmetrical 180 degree rotations in the solvent but not in the complex - which merited a symmetry correction in our final FEP prediction for this compound.

The resolution of a prediction outlier

In an initial run of the HIF-2α perturbation map, ligand 35 (panel A of Supplementary Figure 4) was predicted to bind 4 kcal mol^{-1} more strongly than experimental measurement. In the patent that contained the experimental data (US 9,908,845 B2[36]), the experimental IC50 of ligand 35 was 5.7 ± 2.7 μM; relative to the other ligands in the perturbation map, FEP+ predicted ligand 35 to have an IC50 of approximately 5 nM. This (relative) prediction is at the assay detection limit (5 nM) and it is possible that this measurement does not fully account for the experimental uncertainty. The affinities of some of the ligands in the series, including ligand 25, were measured more than once; if one of the measurements was less than the assay detection limit, it was not used in the standard deviation calculation. As a fraction of reported IC50, the standard deviations of the IC50s in the chemical series ranged from 0.0004% to (ligand 86) to 142% (ligand 28).

We sought to understand whether the measured and predicted affinity of ligand 35 was consistent with the structure activity relationship (SAR) with the rest of the series. A small perturbation map was constructed and run that contained

ligand 35 and its closest chemical analogues from the larger map (30, 31, 41, 42, 43, 50). The pairwise RMSE in this small map was 2.2 kcal mol^{-1} but 0.85 kcal mol^{-1} when ligand 35 was removed from the map, suggesting that FEP captures the experimental SAR for chemical analogues of ligand 35, except ligand 35 itself.

To see if the measured affinity of ligand 35 was consistent with the experimental SAR, we searched the patent (US 9,908,845 B2[36]) and a companion paper by Wehn et al.[37] for matched molecular pairs of ligand 35 that were not present in the ligands used by Schindler et al. In the series published by Wehn et al., ligand 7f (panel D of Supplementary Figure 4) the cyano-nitrle (CN) and hydroxyl (OH) groups occupy the same location in the binding site as ligand 35. Ligand 7d (panel E of Supplementary Figure 4) is the same as ligand 7f except that Cl is in the place of the CN group. The pair 7d and 7f indicate that the free energy to convert Cl into CN is +0.53 kcal mol^{-1}. Similarly, matched molecular pairs in the patent (ligands 71 and 72, and 138, 139) imply the conversion of halogen to a CN in the same position has a free energy difference of between +0.1 kcal mol^{-1} and -0.2 kcal mol^{-1}. In contrast, the ligands 35 and 41 (panel B of Supplementary Figure 4) suggest that the free energy to convert Cl to CN (at the same position) is +2.64 kcal mol^{-1}. Ligand 50 (panel C of Supplementary Figure 4) and ligand 35 imply that the free energy of removing OH is +2.56 kcal mol^{-1}. This large difference is inconsistent with matched molecular pairs from the patent (ligands 144 and 146, and 53 and 54), which imply that removing OH from that position changes the binding free energy by between +0.47 and -0.30 kcal mol^{-1}. As the affinity of ligand 35 is substantially different from what is implied by the SAR and related ligands such as 7f, we believe the measurement accuracy to be questionable. Thus, ligand 35 was removed from our perturbation map in the final accuracy evaluation of FEP+.

After removing ligand 35 from the map, the pairwise RMSE was 1.13 ± 0.02 kcal mol^{-1} when the extra rotamer states were ignored and the symmetry corrections were not applied. After applying the binding mode and symmetry corrections, the RMSE was reduced to 1.10 ± 0.02 kcal mol^{-1}.

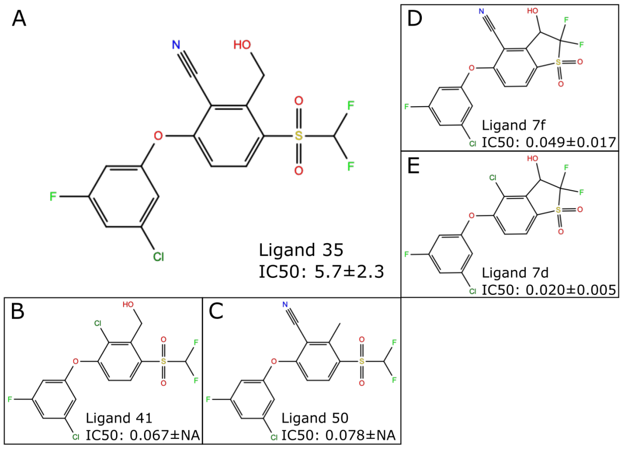

Supplementary Figure 4: Ligand 35 (panel A) was omitted from the set of ligands prepared by Schindler et al. because the measured affinity is substantially inconsistent with estimates from matched molecular pairs shown here. Ligands 45, 41 (panel B), and 50 (panel C) are described in the patent US 9,908,845 B2[36]. Wehn et al. published a companion paper to the patent [Wehn2018Design], which included the ligands 7f and 7d (panels D and E, respectively)[37]. The IC50s are in μM and were measured in a scintillation proximity assay.

PFKFB3

The protein crystal structure that was used by Schindler et al. (PDB code 6HVI) contains three negatively charged molecules (phosphate, fructose 6-phosphate, and citrate) that are buried within the protein. As we suspected that removing these charged molecules would destabilize the nearby protein residues, these charged molecules were retained when repreparing the protein structure. Schindler et al. prepared their system without these molecules.

The ligand structures were initially taken from Schindler et al. and, from visual inspection, it appeared that the binding pocket could support alternate rotamer states of the perturbed R-groups. So each group not in the core of the molecule was manually flipped by 180 degrees and added to the perturbation map. Except for ligands 44, 46, 47, 48, and 49 all ligands had at least one extra rotamer added to the map, increasing the number of nodes in the map from 40 to 87. Ligands 44, 46, 47, 48, and 49 did not require an extra rotamer as the R-groups were symmetrical with respect to 180 degree rotations (see panel C of Supplementary Figure 5). These symmetrical rotations were sampled in the solvent simulations, but not sampled fully in the complex simulations.

Ignoring the new rotamers in the output FEP and using only the nodes with the poses as prepared by Schindler et al., the pairwise RMSE was calculated to be 1.53 ± 0.04 kcal mol^{-1}. After applying the binding mode correction and the symmetry correction for ligands 44, 46, 47, 48, and 49, the pairwise RMSE was reduced to 1.42 ± 0.04 kcal mol^{-1}. The ligand that was impacted most by the additional rotamers was ligand 62 (shown in Supplementary Figure 5), where the $\Delta\Delta G$ between the two rotamers was approximately zero.

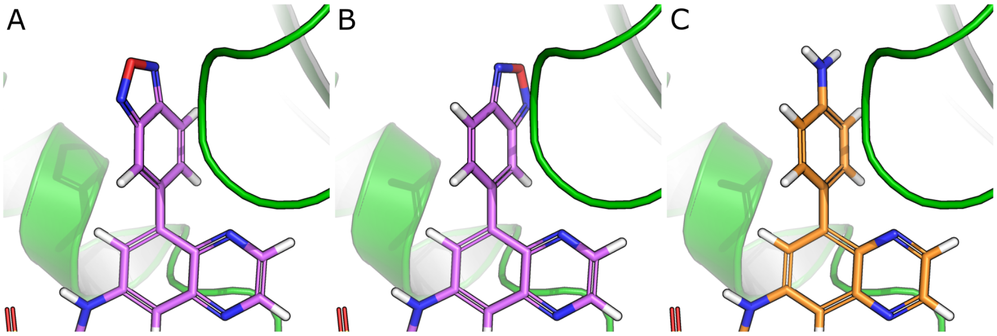

Supplementary Figure 5: Example ligands from the PFKFB3 map. Panel A shows a fused ring of ligand 62 (as labeled in the perturbation map) in the initial state as prepared by Schindler et al. Panel B shows the same ligand but with the fused ring flipped by 180 degrees. FEP+ predicts these rotamers to be approximately equiprobable in the binding site. Panel C shows ligand 49, which has a phenylamine that is symmetrical with respect to 180 degree rotations. This symmetrical rotation is not sampled in the complex simulations but was in solvent.

SHP-2

We used the prepared protein crystal structure (PDB code 5EHR) as well as the same docked ligand poses as Schindler et al. When using the 21-4 release of FEP+, we found the pairwise RMSE in our calculations (1.60 ± 0.06 kcal mol^{-1}) was larger than reported by Schindler et al. (1.24 [1.17, 1.34] kcal mol^{-1}). A large component of this error was driven by a single ligand, called "Example 5" in the perturbation map (see panel A of Supplementary Figure 6), whose relative binding affinity is significantly underestimated. For example, panel B of Supplementary Figure 6 shows Example 1, a close analogue of Example 5 that is also present in the perturbation map. The experimental relative binding free energy from Example 1 to Example 5 is 1.43 kcal mol^{-1}, but FEP+ predicts 3.84 kcal mol^{-1}. When this ligand is removed from the map, the pairwise RMSE is reduced from 1.63 kcal mol^{-1} to 1.43 kcal mol^{-1}. As Supplementary Figure 6 shows, Example 5 has a methoxy group adjacent to ASP 489, which was treated as being deprotonated by ourselves and Schindler et al. To test whether the FEP+ results were sensitive to the protonation state of ASP 489, the FEP+ calculations on this series were repeated with the ASH 489 (i.e. neutral ASP). In these calculations, the pairwise error reduced to 1.22 ± 0.05 kcal mol^{-1}. With ASH 489, the predicted relative affinity between Example 1 to Example 5 was calculated to be 1.44 kcal mol^{-1}, which is very close to the experimental value.

To see if the neutral form of ASP 489 was consistent with our OPLS4 force field, we used our protein residue FEP+ protocol to calculate the pKa of ASP 489 in complex with Example 5, and as a comparison, Example 1. The protein FEP+ calculations were run 50ns with 48 λ windows. Panel C of Supplementary Figure 6 shows the thermodynamic cycle that consists of the two ligands and the protonation states of ASP 489. For the ligand perturbations shown in Panel C of Supplementary Figure 6, the calculations were repeated with ASP 489 in the REST region, without custom charges, and were run with 50ns with 24 λ windows . The direct perturbations between ASP 489 with Example 1 and 5 indicate that the pKa of the ASP is roughly 6, and, as this is below the experimental pH of 7.2[38], this implies that for these ligands, ASP 489 is deprotonated. However, these pKa calculations were inconclusive as the thermodynamics cycle shown in Supplementary Figure 6 has high hysteresis. We suspect that the source of the high hysteresis is related to the conformational sampling of ASP 489 in the Example 1 to Example 5 perturbation. The protein residue FEP+ protocol uses a dual topology approach whereby both the endpoint structures are fully decoupled during the perturbation. In contrast, placing ASP 489 in the REST region only partially weakens its interaction with its environment. An analysis of the $\chi 1$ torsion angles of ASP 489 confirmed this hypothesis (data not shown) and revealed inconsistent conformations between the protein FEP and ligand FEP calculations.

Assuming that the prediction suffers from poor sampling in the ASP Example 1 to Example 5 edge, one can ignore this value and calculate the relative free energy from the remaining edges (colored grey in panel C of Supplementary Figure 6). Doing so produces an estimated relative binding free energy of 2.14 kcal mol^{-1}, which, reassuringly, is close to the experimental value of 1.43 kcal mol^{-1}. Nevertheless, because these ASP 489 pKa calculations suffer from

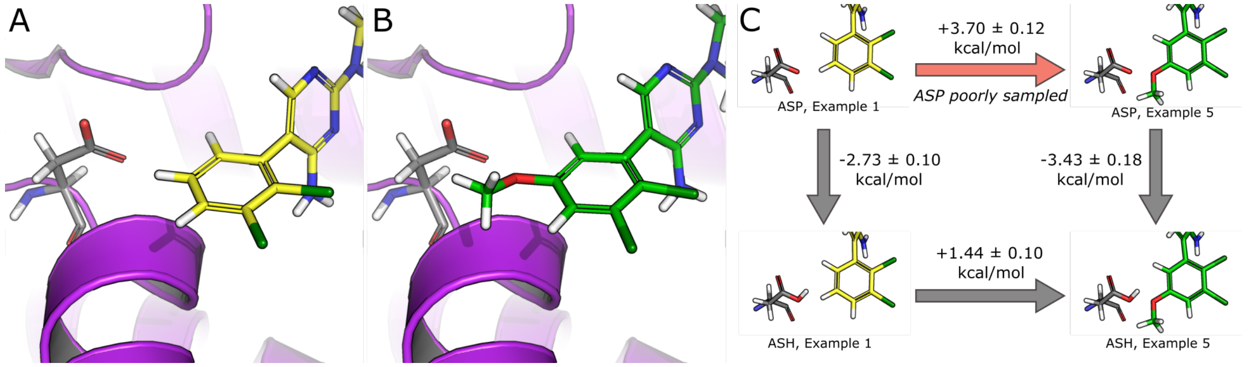

Supplementary Figure 6: An example of poor sampling with SHP2. Panels A and B show ligands Example 1 and Example 5, respectively. The experimental relative binding free energy from Example 1 to Example 5 is 1.43 kcal mol^{-1}, but when ASP 489 (pictured in panels A and B) is deprotonated, the affinity of Example 5 is significantly underestimated relative to other ligands. Protonating ASP 489 (labeled as ASH in panel C) resolves this discrepancy (lower horizontal edge in panel C). However, protein residue FEP+ calculations (vertical edges in panel C) indicate that the pKa of ASP 489 is lower than the pH of the assay, indicating that the deprotonated form of ASP 489 is consistent with our forcefield. Nevertheless, the thermodynamic cycle shown in panel C has high hysteresis (1.56 kcal mol^{-1}) suggesting this conclusion should be viewed cautiously.

high hysteresis, the previously calculated pairwise RMSE of the whole SHP2 map (1.63 kcal mol^{-1}) was used in our benchmark assessment

SYK

The protein crystal structure used by Schindler et al. (PDB code 4PV0) had a partially unresolved P-loop. An alternate crystal structure (PDB code 4PUZ) that also contained a ligand in the same congeneric series was found and prepared for our FEP calculations. LYS 458 adopts a different conformation in 4PV0 than in 4PUZ - the conformation adopted in 4PUZ sterically clashes with some of the ligands in the congeneric series, such as the ligand in 4P0V. Thus, when preparing 4PUZ, the conformation of LYS 458 was changed to be the same as in 4P0V. For the calculations, LYS 458 was included in the REST region.

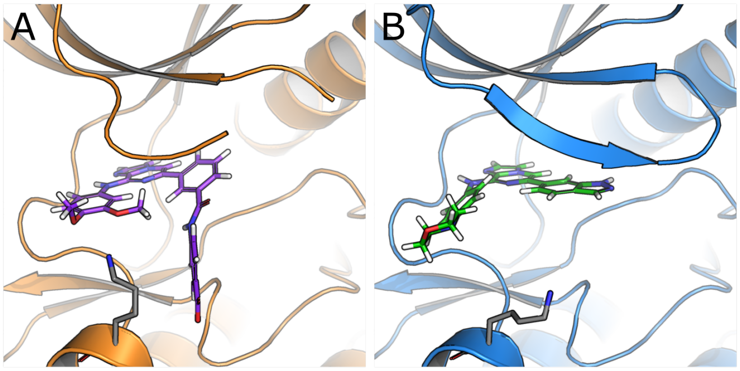

Supplementary Figure 7: Cartoons of the two crystal structures of SYK. Panel A shows the structure (PDB code 4PV0 with ligand 3264995) used by Schindler et al. as the basis for their perturbation map. The P-loop has missing residues which required modeling during the preparation of the protein. Panel B shows the structure used as the basis for our calculations (PDB code 4PUZ, with CHEMBL 3265032). In the foreground of both panels is LYS 458, which was included in the REST region.

This 4PUZ crystal structure indicated that some of the modeled orientations used by Schindler et al. of several ligands could be improved. As a result, several of the ligands were redocked. Additional rotamers were added where ambiguity remained in the orientation of the fused rings. In total, 5 ligands had alternate rotamer states included. To help reduce the size of the perturbations between different ligand groups, two intermediate molecules were added to the map. Epik was used to generate two tautomers of two ligands such that both ligands appeared twice in the perturbation map - the state penalties (as calculated by Epik) for the tautomers was included in the final set of predictions. The ligands that were modified are listed in Supplementary Table 12

Supplementary Table 12: The names of the ligands in the SYK series from Schindler et al. that were redocked and had extra states added to the perturbation graph.

Redocked	CHEMBL3264994, CHEMBL3264998, CHEMBL3264999, CHEMBL32645000, CHEMBL32645004
With extra rotamers	CHEMBL3265011, CHEMBL3265013, CHEMBL3265014, CHEMBL3265015, CHEMBL3265025
With extra tautomers	CHEMBL 3265000, CHEMBL 3265003

With the 4PUZ crystal structure and the redocked ligands, the pairwise RMSE when ignoring the additional tautomers and extra rotamers was 1.13 ± 0.02 kcal mol^{-1}. After correction the tautomers and extra rotamers, the pairwise RMSE was reduced to 1.05 ± 0.02 kcal mol^{-1}.

TNKS2

The same protein structure as used by Schindler et al (PDB code 4UI5) was reprepared starting from the original crystal coordinates. The crystal structure contains a number of partially buried phosphate ions with one that is adjacent to the binding site and makes charged interactions with ARG 977 and LYS 1067. Because of this, the sulfate ions were not removed from the structure in case their removal would destabilize the structure.

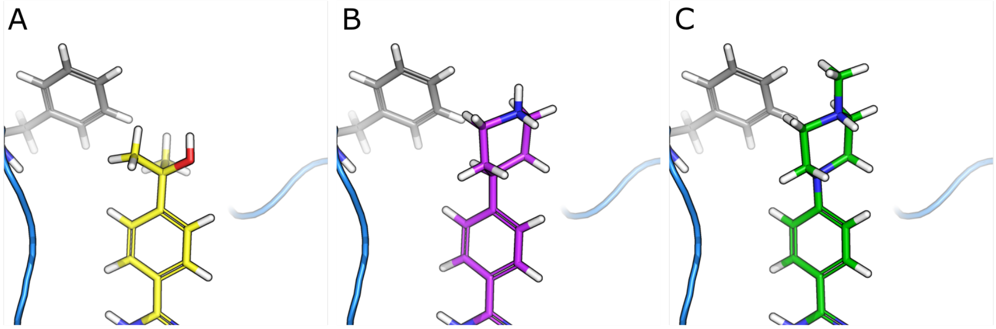

Supplementary Figure 8: Three example ligands bound to TNKS2. Using the ligand names as they appear in the perturbation map, panel A shows ligand 5a, panel B shows ligand. 8d, and panel C shows ligand 8b. PHE 1035 is shown in the background. Ligands 8b and 8d exemplify the six ligands with piperidine or piperazine groups that have predicted affinities underestimated by FEP+. The underestimation is reduced when using an experimental version of OPLS4 that explicitly accounts for cation-pi interactions.

In an initial FEP calculation, the relative binding affinity of six ligands were found to be significantly underestimated by around 2.5 kcal mol^{-1}. The pairwise RMSE in this original FEP was 2.10 kcal mol^{-1}. These six ligands contained positively charged piperidine or piperazine derivatives which were solvent exposed but also surrounded by hydrophobic protein side-chains (see Supplementary Figure 8). Without these ligands, the pairwise RMSE was 0.98 kcal mol^{-1}. The pKa of these six ligands was calculated using Epik[39] and Schrödinger's macropka prediction protocol[40] (shown in Supplementary Figure 8). In case these positively charged groups underwent a significant shift in pKa upon binding, the neutral forms of these ligands were added to the perturbation map. After correcting for the pKa of the ligands using the pH of assay (7.7)[41], the overall RMSE was improved to 1.56 kcal mol^{-1} using the EpiK pKa predictions and 1.61 kcal mol^{-1} using the macropKa pKa predictions. Using the calculated $\Delta\Delta G$s from the perturbation map, the predicted pKa of six ligands when in the complex are shown in Supplementary Table 13.

There are some possible reasons why the six piperidine or piperazine ligands are underestimated by FEP+. The protein loop between PHE 1044 and MET 1054 can change conformation depending on the ligand, as can be seen in the PDB structure 4TJW where the loop responds steric and charged interactions with trimethylamine group that is in the same vicinity as the piperidine and piperazine groups in this map. Although using 4TJW with FEP was not found to reduce the error, it could be that an unknown, alternative conformation can explain the discrepancy of the predictions. A more promising explanation for the underestimation of the piperidine and piperazine groups lies in a possible cation-pi interaction between the charged amines and PHE 1035 (see Supplementary Figure 8). Cation-pi interactions are not explicitly treated in OPLS4 so it is possible that the amine groups are lacking a key interaction in the FEP calculations. Indeed, when the FEP calculations are repeated with an experimental OPLS4 version that includes a cation-pi term, the pairwise error reduces from (roughly)1.6 kcal mol^{-1} to 1.4 kcal mol^{-1}.

Supplementary Table 13: The titratable ligands in the TNKS2 system. The solvent pKas were calculated with both EpiK and Schrödinger's macropka method. The complex pKas have been calculated using FEP and the solvent pKas using FEP+'s pKa correction workflow[42].

	Solvent pKa		Complex pKa	
Ligand name	EpiK	MacropKa	EpiK	MacropKa
8a	8.03	7.82	5.91	5.7
8b	8.03	7.82	5.77	5.56
8c	10.03	10.457	7.1	7.53
8d	10.03	10.447	7.2	7.61
8e	9.47	9.391	6.61	6.53
8f	9.47	9.396	6.74	6.66

2.1.2 FEP+ fragment data set

In 2015, Steinbrecher et al. reported the application of FEP+ on a collection of fragment and fragment-like chemical series[43]. The FEP models were taken from Steinbrecher et al. and modifications to the models were made to the HSP90 and JAK2 data sets, This section details those changes.

Supplementary Table 14: The statistics associated with the FEP graphs in the fragment set. The weighted means have been calculated by weighting edgewise RMSEs by the number of edges in the graph and all other statistics by the number of compounds. Confidence intervals have been calculated by bootstrap sampling over the different graphs.

System	PDB	No. compounds	No. edges	R^2	Edgewise RMSE	Pairwise RMSE
LigA	4CC5	11	14	0.93	0.92 ± 0.24	0.92 ± 0.09
P38	1W7H	6	10	0.90	0.59 ± 0.11	0.56 ± 0.08
T4 lysozyme	186L	12	20	0.43	0.88 ± 0.12	0.90 ± 0.06
Mcl1	4HW3	12	16	0.31	1.26 ± 0.17	1.28 ± 0.10
JAK2 set 1	3E63	10	15	0.76	0.78 ± 0.13	0.87 ± 0.08
JAK2 set 2	3E64	8	13	0.41	1.06 ± 0.16	1.27 ± 0.14
MUP-1	1I06	7	10	0.92	0.48 ± 0.16	0.54 ± 0.07
HSP90 (with pyridine/phenyl moieties)	3FT8	6	8	0.25	1.17 ± 0.23	1.57 ± 0.20
HSP90	3FT8	7	8	0.53	0.60 ± 0.09	0.66 ± 0.08
	Total/weighted mean	79	114	0.59 [0.43, 0.77]	0.92 [0.75, 1.07]	1.01 [0.81, 1.20]

HSP90

HSP90 has a plastic loop region between ASN 105 to ILE 110 that can adopt a variety of conformations depending on the bound ligand. In the original structure used by Steinbrecher et al. (PDB code 3FT8), the region is in a helical conformation which is stabilized by a pyridine group on the ligand. In another structure (PDB code 3FT5) - released along with the original fragment screening manuscript[44] - a small fragment is bound and the flexible region is an 'inward' loop conformation that fills the volume that the pyridine from 3FT8 occupied. The ligands in Steinbrecher's map (from Barker et al.[44]) are in 2 groups: those with an pyridine/phenyl moiety that is buried within the helix (e.g. panel C of Supplementary Figure 9), and those that do not occupy that volume of the pocket (e.g. panel D of Supplementary Figure 9). All the pyridine/phenyl moieties are in the ortho position of a phenyl ring that is consistent between all the ligands in the series. With the smaller fragment in the 3FT5 structure, the volume that would be occupied by the aryl moiety is instead filled by LEU 107 (see panel A of Supplementary Figure 9), so it is possible that this region is filled by the protein when it is bound with the ligands without the pyridine/phenyl moiety.

Initially, a perturbation map was created with both sets of ligands using a newly prepared 3FT8 (helical) structure. In those calculations, it was found that the ligands with the pyridine/phenyl moiety were predicted to bind roughly 2.5 kcal mol^{-1} more strongly (i.e. with higher affinity) than experiment, relative to the ligands without the pyridine/phenyl moiety. This is consistent with the hypothesis that the protein structure from 3FT8 is not in the minimum free energy conformation for ligands exemplified by panel D of Supplementary Figure 9. We expect the offset between the ligands

with and without the pyridine/phenyl moiety to be fully explained by the protein reorganization free energy between these groups of ligands. As any such calculations would be out of the scope of this study, we instead split the map into two separate maps, one for ligands with the pyridine/phenyl moiety and one for ligands without. In first the FEP, the whole pyridine/phenyl moieties were unmapped (in the perturbation region) in order to facilitate rotamer sampling.

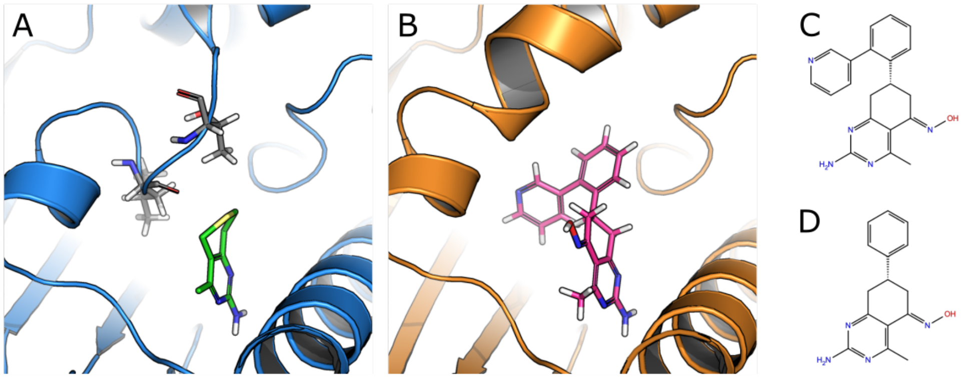

Supplementary Figure 9: The crystal structures and two ligands associated with the HSP90 fragment map. Panel A shows the crystal structure (PDB code 3FT5) with a small fragment bound. The flexible region between ASN 105 to ILE 110 is an inward loop conformation; residues LEU 107 and THR 109 are shown in gray sticks. Panel B shows the crystal structure (PDB code 3FT8) with one of the ligands from the perturbation map bound (ligand 17). The flexible protein region is in a helical conformation. Panel C and D show representative ligands from the HSP90 fragment series; ligands 17 and 9 respectively. If overlaid on the structure shown in panel A, all ligands in this series have a phenyl group that would sterically clash with THR 109 and a subset have an aryl group (e.g. panel C) that would clash with LEU 107.

JAK2

Three protein crystal structures were released with the original medicinal chemistry publication[45] (PDB codes 3E62, 3E63, and 3E64). The structures reveal that the P-loop (residues 857 and 862) can adopt a more closed (e.g. 3E63) or open (e.g. 3E6D) conformation depending on the size of the ligand (see Supplementary Figure 10). The p-loop rearrangement is too large to reliably capture in ligand FEP, meaning that the protein reorganization free energy would not be fully accounted for if all ligands were to be included in a single perturbation map. Because of this, Steinbrecher et al. took 18 ligands from the series by Antonysamy et al. and split them into two perturbation maps, using the more open 3E64 structure as it facilitated ligand docking. Here, we use the same strategy as Steinbrecher et al. and split the ligands into two maps, one map for smaller ligands (those that are the same size as ligand 2 or smaller) and one for larger ligands (those the same size or larger than ligand 13). We refer to the map with the smaller ligands as set 1, and the map with the larger ligands as set 2. Unlike Steinbrecher et al., we use 3E63 for set 1, and 3E64 for set 2, as opposed to 3E64 for both sets.

Both protein structures were re-prepared using the protein-preparation workflow. All crystallographic waters were retained. In the original FEP set 1 map, Steinbrecher et al. had two binding modes for ligand 3 and two tautomers for ligand 4. We retained these alternate states and post processed these using our pka correction (with state populations estimated from Epik) and binding mode correction scripts. All of the ligands from set 2 were redocked using the MCS docking workflow and were manually adjusted. The majority of the ligands in set 2, e.g. ligand 13 in panel B of Supplementary Figure 10, have an internal phenyl group that has two symmetric rotamers. A symmetry correction was not applied for these rotamers as they could partially sample both states in the complex. Ligand 22 has a chlorinated phenyl in that position (see panel C of Supplementary Figure 10), which breaks the symmetry. We added the alternate rotamer of ligand 22 into the map, which was corrected for using our binding model correction workflow. We found the alternate rotamer of ligand 22 to be around 1 kcal mol^{-1} more favorable than the original pose.

2.1.3 GPCRs

This section summarizes our preparation efforts on three GPCR data sets. Two of the systems (A2A and OX2) were adapted from Deflorian et al.[46] and the third (P2Y1) was adapted from Dickson et al.[47].

Although this group of maps only contains three FEP graphs, it is worth considering why all three pairwise RMSEs are above the FEP+ aggregated pairwise RMSE over the whole benchmark. This may be a result of these systems being membrane proteins, as FEP results may be sensitive to membrane setup and relaxation protocol as well as the

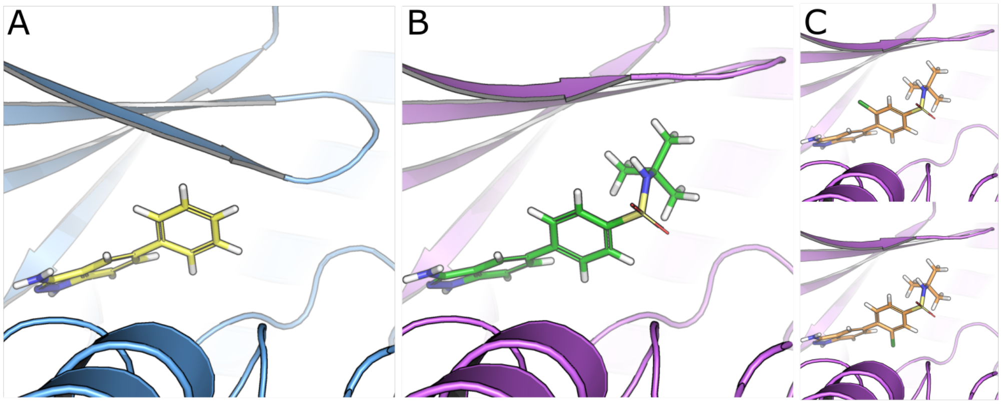

Supplementary Figure 10: Crystal structures and ligand poses associated with the Jak2 fragment maps. Panel A and B shows the crystal structure of JAK2 complexed with ligand 2 (PDB code 3E63) and ligand 13 (PDB code 3E64), respectively. The P-loop adopts a more open conformation for the larger ligand in 3E64. Panel C shows the two chlorinated phenyl rotamers for ligand 22 that were used in the map. The orientation in the lower part of panel C was more favored by roughly 1 kcal mol^{-1}.

composition of the simulated membrane. In all three systems, POPC lipids were used to model the membrane bilayer which may not be approximations to the experimental membrane models. Experimentally, membrane models such as nanodisks and detergent can significantly affect the measured binding affinities[7].

Supplementary Table 15: The statistics associated with the FEP graphs in the GPCR set. The weighted means have been calculated by weighting edgewise RMSEs by the number of edges in the graph and all other statistics by the number of compounds. Confidence intervals have been calculated by bootstrap sampling over the different graphs.

System	No. compounds	No. edges	R^2	Edgewise RMSE	Pairwise RMSE
A2A	17	28	0.50	1.22 ± 0.15	1.58 ± 0.10
OX2	51	103	0.33	1.43 ± 0.09	1.62 ± 0.03
P2Y1	30	76	0.48	1.42 ± 0.10	1.41 ± 0.04
Total/weighted mean	98	207	0.41 [0.33, 0.50]	1.40 [1.22, 1.43]	1.55 [1.41, 1.62]

A2A

This data set originally consisted of 18 ligands split into different perturbation maps. Exemplified in panels A and B of Supplementary Figure 11, each ligand comprises two aryl groups connected to an aminotriazine core. Deflorian et al. prepared seven of the ligands with 2 rotamer states for one of the R-groups. We merged all the ligands and rotamer states into a single perturbation map and excluded one ligand that did not have experimentally measured binding affinity. In our study, we also retained pre-equilibrated protein, solvent, and membrane that was used by Deflorian et al.. Deflorian et al. experimented with different protonation states of HIS 278 (located on the seventh transmembrane helix) and found that the fully protonated form gave better agreement with experiment. In this form, HIS 278 makes a salt bridge with GLU 13 in the first transmembrane helix.

Using Schrödinger's protein mutation FEP+ workflow, the protonation state of HIS 278 was determined for 14 ligands and 6 additional rotamer states. Each protonation state computation comprised the closed thermodynamic cycle between the protonated delta nitrogen (HID), the protonated epsilon nitrogen (HIE), and the fully protonated state (HIP). This closed cycle was generated automatically with the protein mutation workflow. For all protonation state computations, HIP was preferred by between 4 and 10 kcal mol^{-1} compared to either HID or HIE. This strongly implies that HIP is the most appropriate form to use with FEP+ and OPLS4. However, some of these computations suffered from high hysteresis, which was as high as 5 kcal mol^{-1} with one ligand, suggesting that some of these calculations were unreliable. Visual inspection of the complex trajectories revealed that when HIS 278 was in either the HIE or HID forms, the distance between GLU 13 and HIS 278 could increase to over 10 Å, which was accompanied by separation between the first and seventh transmembrane helices. Supplementary Figure 11 shows an instance of this increased separation. The larger separation between HIS 278 and GLU 13 and helix separation was not observed with the HIP form. Thus, it seems that the fully protonated form of HIS 278 is required for structural stability. A mutagenesis study

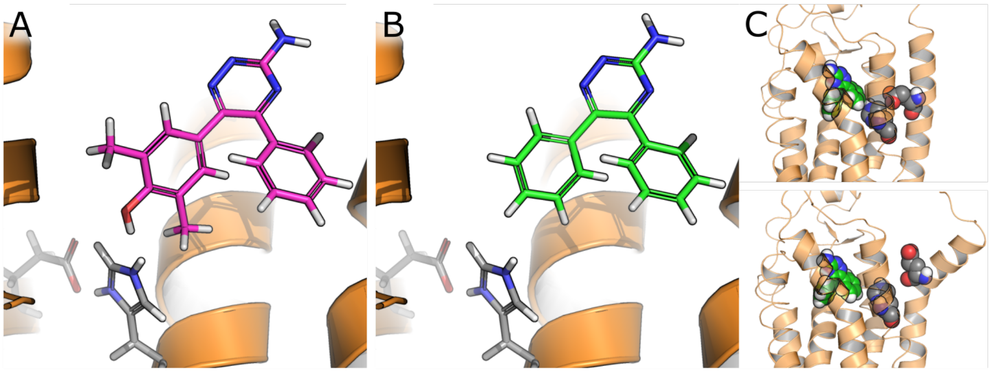

Supplementary Figure 11: Example ligands and structures from the A2A series from Deflorian et al. Panels A and B show ligands 4f and 4a, respectively, with HIS 278 and GLU 13 as gray sticks in the background. The OH of the dimethylphenol group in panel A is predicted as being deprotonated in the presence of the fully protonated, positively charged, form of HIS 278 (see Supplementary Table 17). The relative affinity of ligand 4a was underpredicted by FEP+, which may be due to interactions and conformations that are not well represented by a single binding mode or site. Panel C shows example conformations of the A2A-ligand 4a complex when HIS is in the epsilon protonated form (HIE) taken from a protein FEP mutation simulation. The atoms in the ligand, HIS 278, and GLU 13 are shown as spheres. The top image of panel C shows the structure at the start of a simulation and the lower image shows the structure after 5 ns. The distance between HIS 278 and GLU 13 has increased and the first and seventh helices have partially separated. The protein structure is stable when HIS 278 is fully protonated.

pointed to the importance of HIS 278 and GLU 13 for agonist binding[48], which also hints at the structural importance of maintaining a salt bridge between these two residues. For these reasons, the fully protonated form of HIS 278 was used in our protein-ligand FEP study.

All ligands in the series had at least one aryl group with two degrees of rotational symmetry and ten ligands (shown in Supplementary Table 16) had rotational symmetry in both aryl groups. These degrees of symmetry were not sampled in the complex but were in solvent. This meant that the ten ligands with 2 symmetric aryl groups required a symmetry correction. The ligands that had two rotamers in the perturbation map are also shown in Supplementary Table 16.

Supplementary Table 16: The ligands in A2A had either two rotamers or required a symmetry correction.

Ligands with 2 rotamer states	4b, 4e, 4j, 4k, 4l, 4m, 4r.
Ligands that required a symmetry correction	4a, 4c, 4d, 4f, 4g, 4h, 4i, 4n, 4o, 4q.

Three of the ligands (4e, 4f, and 4o) in these series contain a phenol group that was modeled as being adjacent to HIS 278. As shown in Supplementary Figure 11, the close proximity of the OH group to the positively charged histidine suggests that this group could be deprotonated when bound to the protein. As a result, the deprotonated forms of 4e, 4f, and 4o were added to the perturbation map. The phenol group of ligand 4e is chlorinated in the meta position and was modeled by Deflorian et al. in two orientations, both orientations had deprotonated analogues added to the perturbation map. The pH of the assay was 7.5[49] and the solvent pKas for 4e, 4f, and 4o were estimated using Epik. After the FEP+ calculations were completed, the pKa tautomer correction script was used to compute the contribution of these additional protonation states to the relative binding free energies. Supplementary Table 17 shows the EpiK calculated pKas and the calculated pKas when bound to A2A; ligands 4f and 4o are predicted to predominantly be deprotonated (i.e. negatively charged), whereas ligand 4e is predicted to be protonated (i.e. neutral) for both of its conformations.

It should be noted that the pKa shifts of ligands 4f and 4o have been calculated under the assumption that HIS 278 is fully protonated. This assumption could be valid if deprotonating HIS 278 to either HIE or HID incurs significant protein reorganisation free energy penalties, as suggested with the protein FEP calculations.

After applying the symmetry, binding mode, and pKa corrections (in that order) to the output from FEP+, the pairwise RMSE was 1.58 ± 0.10 kcal mol^{-1}. The ligand with the largest error is 4a - removing this ligand from the perturbation map reduced the pairwise error to 1.35 ± 0.09 kcal mol^{-1}. The relative affinity of ligand 4a is underestimated by roughly 2 kcal mol^{-1}. Only the aminotriazine core of ligand 4a is hydrogen bonded to the receptor, otherwise its interactions are hydrophobic and shape-based. The affinity for the ligands in this series were measured using SPR that used A2A embedded in a micelle attached to the detection chip[49]. Unlike other ligands in this series, the association and dissociation of 4a is very fast and beyond the time resolution of the experiment. This observation could be explained if

Supplementary Table 17: The pKa of 3 ligands that contain a phenol group adjacent to HIP 278. The solvent pKas were calculated with EpiK and the relative free energy to deprotonate the OH group of the phenol was calculated with the protein mutation workflow of FEP+. The calculated pKa when bound to A2A is shown in the final column. Ligand 4e was modeled by Deflorien et al. in two orientations: “up” and “down”.

Ligand name	Solvent pKa (EpiK)	$\Delta\Delta G$ to deprotonate (kcal mol^{-1})	Complex pKa
4e up	8.43	3.55 ± 0.20	11.01
4e down	8.43	2.19 ± 0.35	10.03
4f	10.72	-5.63 ± 0.42	6.62
4o	9.84	-3.80 ± 0.42	7.07

4a were to bind weakly in multiple ways to the A2A-micelle system. As SPR detects changes in mass, the detection of a binding event could result if a ligand were to bind to the micelle and not the protein. A more rigorous approach to predicting binding affinity to this membrane-micelle system would also include the possibility of the ligand binding to the micelle, similar in spirit to the recent study by Dickson et al.[47]. In case the underestimation of ligand 4a was due to missing relevant binding modes, the entire pose was rotated by 180 degrees along the axis formed between the two aryl groups. This maintained the same binding site shape complementarity and hydrophobic interactions at the expense of weaker hydrogen bonds in the core. However, this flipped binding mode was roughly 2 kcal mol^{-1} less favorable than the original pose. The pairwise RMSE of 1.58 kcal mol^{-1} was used in our aggregate accuracy assessment.

OX2

As with A2A, the protein ligands structures were taken from the publication by Deflorien et al.[46]. Originally, the OX2 ligands were divided into two sets and separate FEP maps, each of which focused on perturbations on two different regions of the ligand scaffold. Here, we opted to create a single map that contained all the ligands.

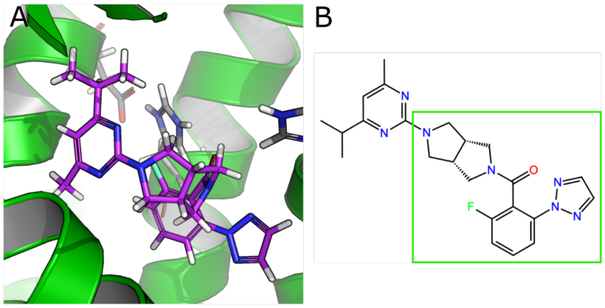

Supplementary Figure 12: Panel A shows ligand 1 from set 1 bound to OX2. In the background are ASP 66 and HIS 265 which potentially form a salt bridge between two transmembrane helices. Panel B shows the 2D structure for ligand 1 from set 1. The atoms within the green box made up the custom core and the pyrimidine group outside the custom core was decoupled in the intermediate lambda windows. This was found to enhance sampling of the dihedral between the pyrimidine group and the custom core in the complex and in solvent.

Visual inspection of the binding modes of the ligands revealed that the orientation of a pyrimidine group could be fit in the binding site in two possible orientations. The addition of multiple rotamers in the perturbation map was explored for this system, but it was found that the orientation did not interconvert in the solvent legs, which violates an assumption of the binding mode correction. Instead, a custom core was created that decoupled the pyrimidine group in the intermediate lambda windows. To further facilitate sampling of the pyrimidine group, the torsion potentials were also scaled to zero in the intermediate lambda windows (using the ‘modify-dihe’ option of FEP+). After the completion of the FEP calculations, rotations of the pyrimidine group were observed in the complex and in solvent.

Previously, Deflorian et al. investigated the accuracy of FEP with OX2 with different protonation states of HIS 175 and 265 (as numbered in the structure and referred to as 224 and 350 in manuscript). They found improved accuracy with using the fully protonated forms of both histidines. HIS 265, in particular, potentially forms a salt bridge with ASP 66 and both are on two adjacent transmembrane helices. As HIS 265 is in close proximity to a perturbation site of the ligand, FEP calculations were considered to predict the pKa of this residue. However, given the difficulty in the HIS pKa calculations in A2A, we decided to use the fully protonated form of this residue, as well as with HIS 175.

The pairwise RMSE on this system was 1.63 ± 0.03 kcal mol^{-1}. When calculating the error separately of the two ligand sets of Deflorian et al., set 1 had a pairwise RMSE of 1.67 ± 0.05 and set 2 had a pairwise RMSE of 1.25 ± 0.05 kcal mol^{-1}.

P2Y1

The P2Y1 purinergic receptor is a class A 7-transmembrane GPCR activated by ADP and able to trigger the release of glutamate in the thrombus formation process[50]. This mechanism can be altered by the binding of the allosteric antagonist BPTU to the transmembrane region of P2Y1, as highlighted by the reduction of platelets aggregates in the analyzed samples[51]. Following the work by Dickson et al.[47], we studied 30 antagonists reported by BMS from the same chemical series of BPTU[51] starting from the crystal structure of the complex P2Y1-BPTU (PDB code 4XNV)[50]. The protein structure was obtained from the Orientation of Proteins in Membrane database[52] and prepared with the Protein Preparation Wizard from the Schr odinger suite. Ligprep was used to prepare the ligands, and Glide with core constraints based on BPTU was used to dock the ligand chemical series onto the P2Y1 receptor. Membrane and solvent were added to the system by the FEP+ panel in Maestro, which was also used to build the perturbation map and generate the input files for initial and exploratory FEP calculations using default simulation parameters (12λ windows, 5ns). The results obtained showed a R^2 of 0.47 and pairwise RMSE of 1.58 kcal mol^{-1}. To improve inadequate R-group sampling, a custom core on the central pyridine and amide groups (atoms in the green square in Supplementary Figure 13) was used in following FEP runs with 24λ and 20ns as simulation length. In addition, the ligands were divided into two maps according to the presence of substituents attached to the ortho or meta position on one of the benzene rings (ring in the cyan square in the image), and the two maps were merged to obtain the final binding free energy predictions. These changes resulted in a R^2 of 0.48 and pairwise RMSE of 1.41 kcal mol^{-1}.

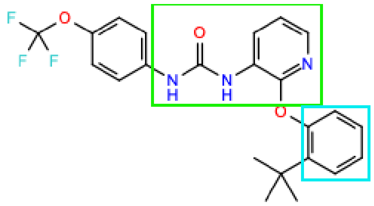

Supplementary Figure 13: The atoms within the green square were used to define a custom core, the benzene ring surrounded by the cyan square was selected to divide ligands into two groups to and run a separated FEP+ map for each group. The first group of ligands presented an ortho substituent attached to the benzene ring in the cyan square, the second group was formed by all the ligands with a meta substituent attached to that benzene ring plus ligand 16 and ligand 11a. The presence of these last two ligands allowed the merging of the two FEP+ calculations into a single one after their completion for ΔG calculations.

2.1.4 Janssen BACE1 data sets

In two publications[53;54], the pharmaceutical company Janssen published two papers on the development of BACE1 inhibitors whose design was guided by FEP+.

Supplementary Table 18: The FEP+ statistics of the Janssen BACE 1 set. The weighted means have been calculated by weighting edgewise RMSEs by the number of edges in the graph and all other statistics by the number of compounds. Confidence intervals have been calculated by bootstrap sampling over the different graphs.

System	PDB	No. compounds	No. edges	R^2	Edgewise RMSE	Pairwise RMSE
Keränen et al., P2	3IN4	12	18	0.07	0.59 ± 0.09	0.59 ± 0.05
Keränen et al., P3	3ZOV	21	36	0.63	1.28 ± 0.16	1.55 ± 0.06
Ciordia et al., retrospective	4JPC	32	61	0.55	1.06 ± 0.09	1.16 ± 0.03
Ciordia et al., prospective	4JPC	9	12	0.59	1.10 ± 0.37	1.19 ± 0.18
	Total/weighted mean	74	127	0.50 [0.27, 0.62]	1.08 [0.83, 1.24]	1.22 [0.90, 1.45]

Ciordia et al. 2016

Ciordia et al. used FEP+ to prospectively select chemical modifications to a BACE1 series[53]. Prior to the prospective calculations, Ciordia et al. validated FEP+ using a retrospective set of compounds. After running FEP prospectively,

proposed compounds were then synthesized and assayed, and the calculated free energies were found to agree well with the measured relative activities. Although the retrospective and prospective set of compounds could be included in a single perturbation map, the sets were run separately in order to preserve the original structure of the study.

The retrospective set:

This series primarily explores R-group perturbations to a terminal phenyl group. The crystal structure (PDB code 4JPC) was downloaded and prepared using the protein preparation wizard. All crystallographic waters were retained. The ligand binding modes were downloaded from the supporting information of the publication[53]. In the first iteration of the perturbation map, extra rotamers that could be sterically accommodated in the binding site were added to the perturbation map. However, some of these extra rotamers were found to flip back and forth between both orientations; for the binding mode correction to be correctly applied, the binding modes must not be able to interconvert. Instead, a new perturbation map was created using a custom core that excluded the perturbation region of the ligand. To check if this sufficiently facilitated sampling about the torsion angle of interest, the dihedral distributions of the aryls that have rotameric symmetry were analyzed. These were found to have correctly sampled both rotameric states with approximately equal probability in the complex and the solvent.

Ligand 30 has a pyridine ring that was modeled as being neutral. EpiK predicts the pKa of the piperidine nitrogen to be 6.45, implying that, depending on the pH of the assay, the protonated form could be a significant population in solvent. The pH of the assay was not reported, but BACE1 is primarily active between pHs of 4 and 5[55]. When only the neutral form was modeled in the map, the binding free energy of ligand 30 was predicted to be too negative by 1.7 kcal mol^{-1}. Adding the protonated form of ligand 30 and applying the pKa correction (assuming a pH of the assay was 4.5) reduced the relative error of this ligand to 1.2 kcal mol^{-1}.

When the protonated form of ligand 30 was ignored, the overall pairwise RMSE was 1.19 ± 0.04 kcal mol^{-1}. Including the protonated form of ligand 30 and using the pKa correction modestly reduces the overall pairwise RMSE to 1.16 ± 0.03.

The prospective set:

The same protein structure was used for this smaller set of twelve ligands as for the retrospective set. The series explores heterocycle and R-group modifications to a terminal aryl group. The binding modes were taken from the study by Ciordia et al. in which the R-group perturbations extend into the so-called P3 pocket of BACE1. Long chemical groups such as cyano-nitrile and 2-butyne were primarily modeled as growing into the P3 pocket. Although these could plausibly be modeled as also pointing towards the solvent, these additional orientations were not added to the perturbation map. Two ligands have symmetric R-groups (spiro2 and spiro3) but, after completion of the FEP+ simulations, the symmetric rotamers were sampled equally well in solvent and in the complex. The pairwise RMSE for this system and set of ligands was 1.19 ± 0.18 kcal mol^{-1}.

Keränen et al., 2017

In the two crystal structures that were used for Keränen et al.'s FEP calculations (PDB codes 3IN4 and 3ZOV)[54], there are small structural rearrangements of the protein between the sets of ligands that explore either pocket (for instance, the backbone of GLY 73 and the side chain of TYR 132). Because of this, we used the same strategy of Keränen et al. and divided the ligands into two perturbation maps. We used the 3IN4 protein structure for the P2 pocket perturbations and the 3ZOV structure for the P3 pocket perturbations. Both crystal structures were reprepared using the protein preparation wizard. All crystallographic waters were retained.

The P2 pocket The P2 pocket series explores different R-group perturbations to a methoxyphenyl group in the meta position. Two rotameric states exist for most of these perturbations, either facing towards the protein ("in") or facing towards bulk solvent ("out"). For ligands 30 to 35, the perturbation R-groups are too large to fit in the "in" position. Previously, Keränen et al. used FEP and agreement with experiment to preselect which rotamer should be used for the relative binding free energy calculations. Here, we choose an approach that can be applied in prospective cases and add both rotamers to the map and post process using the binding model correction script. Interestingly, for the majority of the orientations, the "out" conformation is predicted as being more favorable (see Supplementary Table 19). Visual inspection of the simulations reveals that the "out" conformations are stabilized by a water molecule that binds in between the protein and ligand.

When the output perturbation is processed using the rotamer stares used by Keränen et al. and ignoring the additional rotamers, the pairwise RMSE was 0.91 ± 0.07 kcal mol^{-1}. When processing all rotamers with the binding mode correction script, the pairwise RMSE was reduced to 0.59 ± 0.05 kcal mol^{-1}.

Supplementary Table 19: The BACE ligands from Keränen et al. that explore perturbations to the P2 pocket. The R-groups can be modeled as being partially buried in the protein ("in") or facing bulk solvent ("out"). The Supplied Pose column shows which orientation was preselected by Keränen et al., the Poses simulated column shows which orientations were included in our perturbation map, and the final column shows the (cycle closure corrected) $\Delta\Delta G$ between the "in" and "out" pose. All poses were automatically corrected for using the binding mode correction script.

Ligand name	Supplied pose	Poses simulated	$\Delta\Delta G$ in $\rightarrow$ out
28	In	In and out	$-0.75 + / - 0.06$
29	Out	In and out	$-0.23 + / - 0.08$
30	Out	Out	-
31	Out	Out	-
32	Out	Out	-
33	Out	Out	-
34	Out	Out	-
35	Out	Out	-
36	In	In and out	$-1.21 + / - 0.20$
37	In	In and out	$-1.02 + / - 0.20$
38	In	In and out	$-1.78 + / - 0.15$
39	Out	In and out	$-0.44 + / - 0.51$

The P3 pocket This ligand series consists of acylguanidine ring scaffolds with 5, 6, and 7 members with predominantly hydrophobic substituents that are placed in the vicinity of GLU 400 and acylguanidine ring scaffolds with 5, 6, and 7 members. As described below, this series represents a challenge for FEP as the protonation state of GLU 400 is predicted to be dependent both on the ligand and on the conformation of ARG 368.

The crystal structure used for this study (PDB code 3ZOV) has ligand 17e bound, which has a 6 membered ring in the scaffold and a trifluoromethyl substituent within 5 Å of the carboxylate of GLU 400. In contrast, the terminal carbon of the largest R-group substituent, 2-butyne of ligand 17g, is modeled as to be within 3 Å of the carboxylate of GLU 400. Although this steric clash is resolvable during the minimization stages of FEP+, the close proximity of hydrophobic groups to the carboxylate of GLU 400 was expected to affect the pKa of the side chain.

In the 3ZOV crystal structure, the side chain of ARG 368 has 50% occupancy when in an inward orientation that makes a salt bridge with GLU 400, and 50% occupancy facing towards solvent. We expected the pKa of GLU to be lower when ARG 368 is in the inward conformation compared to when ARG 368 is in the solvent facing conformation. With ARG 368 starting in the solvent facing position, pKa calculations were performed on GLU 400 for every scaffold and R-group substituent (21 calculations in total). With ARG 368 starting in the inward orientation, pKa calculations of GLU 400 were performed for the seven R-group substituents of the 6 membered ring in the scaffold. In these calculations, ARG 368 was added to the REST region to aid its conformational sampling.

Supplementary Table 20 shows that predicted pKas for GLU 400 are dependent on the starting conformation of ARG 368 despite the fact that it was added to the REST region. Given this starting structure dependency, these calculations were unable to *prospectively* predict the final protonation state of GLU 400. Out of the 21 calculations performed with ARG 368 starting in the solvent facing conformation, the lowest predicted pKa was 5.5 (with ligand 8a) and the highest was predicted as 9.05 (with ligand 8e). The average over all three scaffold ring sizes is consistent with the pKa calculated with only the 6 membered ring. When ARG started in the inward conformation, the pKa for GLU 400 was found to range from 4.4 to 5.7. Although the publication Keränen et al. did not report the pH of the enzymatic FRET assay, BACE1 has a peak activity with a pH of around pH 4.5[55]. Thus, although we do not know the exact pH of the assay, it is highly likely that GLU 400 is protonated and neutral when ARG 368 is in the solvent facing conformation. When ARG 368 is in the inward conformation, the protonation state of GLY 400 is uncertain and may contain significant populations of both protomers.

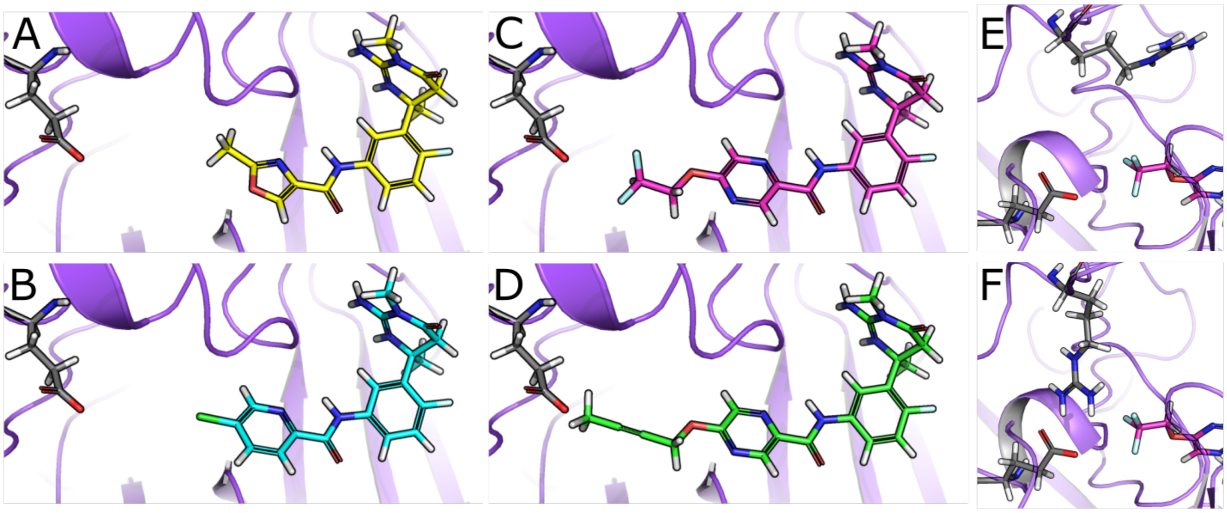

Supplementary Figure 14: Panels A through D show example R-group substituents on the 6 membered acylguanidine ring scaffold. GLU 400 is shown with gray sticks. In the naming scheme of Keränen et al., panel A shows ligand R-group *a*, panel B shows R-group *b*, panel C shows R-group *e* (crystallized in 3ZOV), and panel D shows R-group *g*. Panel E shows ARG 368 in the solvent facing outward conformation, and panel F shows ARG 368 in the inward conformation.

Supplementary Table 20: Predicted pKas of the sidechain of GLU 400 when the simulations are started with two different conformations of ARG 368. In the “in” conformation, ARG 368 and GLU 400 can form a salt bridge; ARG 368 is in the solvent for the “out” conformation. The pKa was calculated for all ligands in the map when ARG 368 started in the outward conformation, and only for the 6 membered scaffold ring when ARG 368 started in the inward conformation. The Bennett Acceptance Ratio errors are shown for the 6 membered ring results, and the average over the three scaffolds is shown with standard error across the 3 scaffolds.

	ARG 368 out		ARG 368 in
Ligand substituent	pKa with 6 membered ring	pKa averaged over 5, 6, and 7 membered rings	pKa with 6 membered ring
a	5.82 ± 0.10	5.70 ± 0.10	4.63 ± 0.10
b	6.02 ± 0.09	5.83 ± 0.26	4.41 ± 0.10
c	6.88 ± 0.11	6.98 ± 0.13	5.06 ± 0.10
d	6.29 ± 0.12	6.83 ± 0.29	4.78 ± 0.10
e	8.91 ± 0.12	8.67 ± 0.31	5.71 ± 0.09
f	7.75 ± 0.12	7.41 ± 0.17	4.93 ± 0.10
g	7.49 ± 0.14	8.03 ± 0.27	4.97 ± 0.11

Based on these pKa calculations, two ligand FEP maps were created. One with ARG 368 in the solvent facing conformation with the protonated form of GLU 400 (referred to below as “GLH 400, ARG 368 out”), and the other with ARG in the inward conformation (referred to below as “GLU 400, ARG 368 in”). ARG 368 was added to the hot region for both perturbation maps. Unlike Keränen et al., we included all R-group perturbations and scaffold hopping perturbations in the same map. Preliminary results revealed poor replica exchange between the lambda replicas for these scaffold hopping calculations. Thus, in these calculations, we used 32 lambda windows for the scaffold-hopping transformations, 24 lambda windows for the R-group transformations, and 40ns of simulation time per replica.

The pairwise RMSE for the “GLH 400, ARG 368 out” map was 2.10 ± 0.10 kcal mol^{-1} and the pairwise RMSE for the “GLU 400, ARG 368 in” map was 1.89 ± 0.07 kcal mol^{-1}. To help determine whether these high errors were due to the R-group substituents, which are in the vicinity of GLU 400, or the scaffold hopping transformations, the perturbation map was divided into three submaps, consisting exclusively of the 5, 6, and 7 membered scaffold rings. Supplementary Table 20 shows the pairwise RMSE of the submaps formed and that the “GLU 400, ARG 368 in” perturbation submaps consistently have substantially lower RMSEs than the submaps with “GLH 400, ARG 368 out”. Retrospectively, this provides evidence that, at least with OPLS4, ARG 368 is found predominantly in the inward conformation with GLU 400 deprotonated. These results also indicate that the high error with the “GLU 400, ARG 368 in” map is driven by the

scaffold-hopping transformations. Indeed, the edgewise error between the different sized rings is approximately 1.87 kcal mol^{-1}.

Supplementary Table 21: The pairwise RMSEs of ring size sets with different conformations of ARG and protonation states of GLU

Size of ring in scaffold	GLH 400, ARG 368 “out”	GLU 400, ARG 368 “in”
5	1.96	0.9
6	2.06	1.49
7	2.31	1.24

2.1.5 Bayer macrocycles

Wagner et al. evaluated the performances of FEP+ macrocyclization protocol of 5 small data sets[56]. One data (ALK) was excluded because 2 out of the 3 compounds were had affinities beyond the experimental limit, another data set (MTH1) was excluded because it was the same as the data set used in our other macrocycle evaluation[57], another data set (FVIIa) was found have binding affinities from racemic mixtures[58]. This left 2 data sets, FTase and BRD4. Using default settings, the FEP+ predictions were found to be accurate with BRD4, whereas the results for FTase required further scrutiny, as described below.

Supplementary Table 22: The summary of the FEP graphs in the Bayer macrocycle set and their associated accuracy metrics. The weighted means have been calculated by weighting edgewise RMSEs by the number of edges in the graph and all other statistics by the number of compounds. Confidence intervals have been calculated by bootstrap sampling over the different graphs.

System	PDB	No. compounds	No. edges	R^2	Edgewise RMSE	Pairwise RMSE
Ftase	1LD7	5	8	0.15	0.64 ± 0.08	0.70 ± 0.12
BRD4	5UEX	3	3	0.90	1.00 ± 0.28	1.00 ± 0.28
	Total/weighted mean	8	11	0.43 [0.15, 0.90]	0.75 [0.64, 1.00]	0.82 [0.70, 1.00]

Ftase

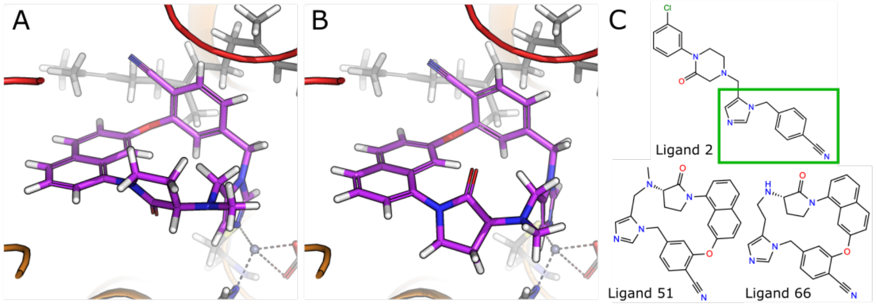

Supplementary Figure 15: The ligands and example binding modes for the Ftase macrocycle system by Wagner et al.[56]. Panels A and B show two model binding modes for ligand 51 - in panel A is the modeled pose by Wagner et al. and in panel B is the binding for ligand 51 as suggested by the crystal structure from the PDB entry 1LD8 that was used in this study. Panel C shows the 3 original ligands used by Wagner et al. The custom core that was used in the FEP+ calculations is highlighted by the green box; all atoms outside that green box were unmapped in the perturbations.

This chemical series, originally by Bell et al.[59], consists of three ligands: two macrocycles (ligands 51 and 66) and one acyclic molecule (ligand 2). The binding site contains a cofactor that has a consistent pose in the three crystal structures that contain two ligands from the series (PDB codes 1S63, 1LD7). We used the same protein structure as Wagner et al. (1LD7) but reprepared it from scratch. Another crystal structure, PDB code 1LD8, had a ligand bound that is a close chemical analogue of ligand 51. As shown in Supplementary Figure 15, this crystal structure suggested that the pose used by Wagner et al. for ligand 51 could be improved, particularly regarding the orientation of the gamma-lactam moiety; accordingly, the modeled pose of ligand 51 was adjusted.

Using the default FEP+ mapping and simulation settings, the initial FEP+ calculations were found to have extremely high hysteresis - at around 8 kcal mol^{-1} - and the pairwise RMSE was 2.45 kcal mol^{-1}. It was hypothesized that the high hysteresis was a result of inconsistent atom mappings between the 3 ligands. In one macrocyclization edge, a large number of atoms were mapped between a macrocycle (51) and the acyclic ligand (2), whereas in the other macrocyclization edge, the entire linker region was left unmapped. To resolve the hysteresis, a custom core was applied to the map that left the entire linker unmapped for all edges. The custom core that was used is shown in panel C of Supplementary Figure 15. To facilitate the sampling of the linker dihedral angles, the torsion angle barrier heights were scaled to zero in the intermediate lambda windows. Additionally, the ligands - that were initially modeled as being neutral - had protonatable amines. Extra protomers were added to the map but were predicted by FEP+ to be very unfavorable and were neglected in subsequent calculations.

Using the custom core, the pairwise RMSE was reduced to 0.90 +/- 0.23 kcal mol^{-1}. However, one macrocyclization edge (between ligands 2 and 51) was found to have a high estimated Bennett Acceptance Ratio (BAR) error of 0.44 kcal mol^{-1}, which is indicative of insufficient sampling, or low overlap between the lambda windows, or both. Thus, the simulations were repeated with 48 lambda windows run for 40ns each (compared to the default of 16 lambda windows run for 20ns each). This reduced the estimated BAR errors in that edge to an acceptable 0.12 kcal mol^{-1}. The pairwise RMSE for the 3 ligands in these extended calculations was 0.61 +/- 0.18. Finally, the series by Bell et al. contained 2 close chemical analogues of ligands 51 and 66 that differed only in the methylation of an amine[59]. These two analogues were added to the perturbation map. With these ligands in the FEP+ map (run with more lambda windows and longer simulation times) the pairwise RMSE was 0.70 +/- 0.12 kcal mol^{-1}. This value is used in the RMSE aggregate analysis.

2.1.6 MCS docking sets

These data sets were taken from the work by Cappel et al.[60] which developed a combined maximum common substructure docking procedure for use with FEP. Other data sets that were used by Cappel et al., were not used in this study as comprised of targets that were already present in this benchmark assessment.

Supplementary Table 23: The statistics associated with the FEP graphs in the MCS docking sets. The weighted means have been calculated by weighting edgewise RMSEs by the number of edges in the graph and all other statistics by the number of compounds. Confidence intervals have been calculated by bootstrap sampling over the different graphs.

System	PDB	No. compounds	No. edges	R^2	Edgewise RMSE	Pairwise RMSE
Renin	3OOT	29	47	0.46	1.80 ± 0.14	1.50 ± 0.04
HNE	5A0A	20	30	0.74	1.37 ± 0.17	1.39 ± 0.06
	Total/weighted mean	49	77	0.57 [0.46, 0.74]	1.65 [1.37, 1.80]	1.46 [1.39, 1.50]

Renin

The ligands in this series primarily consist of R-group perturbations to 2 aryl groups and additions to an indole core. As the R-group additions to the indole include alcohols and are adjacent to HIS 287, the tautomeric populations of HIS 287 may differ between the ligands. The same protein structure as Cappel et al. was prepared (PDB code 3OOT) using the standard workflow. PropKa predicted HIS 287 to be in the epsilon tautomer (HIE), which is in contrast to Cappel et al., who used the delta tautomer (HID). Two perturbation maps were created with the two neutral HIS 287 tautomers. The binding modes were taken from the previously prepared MSC docking set. As other docking methods predicted alternative orientations of an aryl group, a custom core was created in FEP+ that left this group unmapped between perturbations. To facilitate dihedral rotations of this aryl, the torsion energies between the aryl and scaffold were scaled to zero in the intermediate lambda windows using the ‘modify dihedrals’ option of FEP+.

Upon completion of the simulations, the pairwise RMSE was 1.50 ± 0.04 kcal mol^{-1} for the map with HIE 287 and 1.57 ± 0.05 for the map with HID 287. However, this error was not found to be significantly affected by the R-group additions to the indole core which alter the interaction with HIS 287. Removing the ligands with the indole additions from the maps resulted in pairwise RMSEs of 1.53 ± 0.07 kcal mol^{-1} for HID 287 and 1.49 ± 0.07 kcal mol^{-1} for HIE 287. As a result, the prediction of the tautomeric states of HIS 287 was not pursued further.

The experimental assay conditions for this study are included in the patent that accompanied this chemical series[61]. Interestingly, bovine serum albumin (at a concentration of 1.25 mg/ml) was present in the assay solution. Serum albumin a promiscuous binder of small molecules and its presence in the assay may contribute to the error in this series. The pairwise RMSE of the whole HIE 267 map was used in our benchmark aggregate RMSE.

HNE

The ligands in this series bind to a solvent adjacent binding site and the chemical series consists of R-group modifications on two different sites of the scaffold. The ligand poses and prepared protein structure were the same as by Cappel et al.[60].

Different protonation states of three ligands were experimented with in this data set. Ligands 16 and 19 have a carboxylate group as one substituents, which EpiK predicts pKas of roughly 3.6, and ligand 21 has tertiary amine, which Epik predicted to have a pKa of 8.53. As the experimental pH was 7.4[62], ligands 16 and 19 were previously modeled as being deprotonated (negatively charged), and ligand 21 was modeled as being protonated (positively charged). For completeness, the protonated and deprotonated forms of ligands 16, 19, and 21 were added to the perturbation map. When these protomers were processed with the pka tautomer correction workflow, they were found to have no effect on the FEP+ predictions as the pKas did not change substantially.

Upon completion of the simulations, the pairwise RMSE was found to be 1.62 ± 0.08 kcal mol^{-1}. A significant component of the error is due to ligand 6, which experimentally binds to HNE with a ΔG that is at least 3 kcal mol^{-1} higher (i.e. weaker) than the other ligands. FEP+ correctly predicts this ligand to be at the bottom of the assay but predicts it to bind even more weakly than the experiment by roughly 2 kcal mol^{-1}. Removing this ligand from the map reduced the pairwise RMSE to 1.45 ± 0.08 kcal mol^{-1}. This relatively high error prompted revisiting the experimental assay conditions. Interestingly, the assay buffer had a NaCl concentration of 0.5 M, which is significantly larger than other assays in this benchmark. In contrast, FEP+ uses a default NaCl concentration of 0.15 M for charge-changing perturbations and no salt for all others. After repeating the FEP calculations with the same salt concentration of the experiment, the pairwise RMSE reduced to 1.47 ± 0.08 kcal mol^{-1} when the weakest binder was included in the map, and to 1.32 ± 0.07 kcal mol^{-1} without the weakest binder. This series of ligands and protein therefore appears to be somewhat sensitive to the salt concentration. The pairwise RMSE of 1.47 ± 0.08 kcal mol^{-1} (including the weakest binding ligand) was used in our overall benchmark assessment.

2.1.7 FEP+ scaffold-hopping

These data were taken from a previous publication of ours that applied FEP+ to calculations that involve scaffold perturbations[63]. The perturbation maps were taken directly from the original publication without modification.

Supplementary Table 24: The statistics associated with the FEP graphs in the FEP+ scaffold-hopping set. The weighted means have been calculated by weighting edgewise RMSEs by the number of edges in the graph and all other statistics by the number of compounds. Confidence intervals have been calculated by bootstrap sampling over the different graphs.

System	PDB	No. compounds	No. edges	R^2	Edgewise RMSE	Pairwise RMSE
Beta-tryptase	3V7T	2	1	1.00	0.64 ± 0.00	0.64 ± 0.00
Factor Xa	2EI8	3	3	0.96	1.26 ± 0.29	1.25 ± 0.28
EZH2	5IJ7	4	5	0.98	0.20 ± 0.04	0.19 ± 0.04
BACE1	4ZSP	3	3	0.93	0.70 ± 0.20	0.70 ± 0.20
CHK1	3U9N	5	8	0.18	0.81 ± 0.11	0.76 ± 0.11
	Total/weighted mean	17	20	0.73 [0.40, 0.98]	0.78 [0.42, 1.05]	0.77 [0.47, 1.06]

2.1.8 FEP+ macrocycles

The ligands in this subset were taken from the paper by Yu et al.[57], which first validated the macrocyclization functionality of FEP+. We revisited the preparation of two FEP maps: HSP90 (3HVD) and CK2.

HSP90 (3HVD)

This ligand set contains ten ligands originally from a study by Suza et al.[64]. All the ligands except one are macrocycles and they differ solely in the chemical linker. Using the ligands and protein structures from Yu et al.[57], the pairwise RMSE was unacceptably high at $2.52/pm0.19$ kcal mol^{-1}. The high error was exclusively driven by three ligands, namely 16, 17, and 18 (using the numbering from Suda et al.) which had binding free energies that were predicted to be 2.4 kcal mol^{-1} too positive (unfavorable) compared to the experimental value. These three ligands have the same linker that is distinct from the others in this ligand set.

Supplementary Table 25: The statistics associated with the FEP graphs in FEP+ macrocycle set. There are different series for BACE1 and HSP90, these are distinguished by the PDB code that was used for these series in the original publication by Yu et al.[57]. The weighted means have been calculated by weighting edgewise RMSEs by the number of edges in the graph and all other statistics by the number of compounds. Confidence intervals have been calculated by bootstrap sampling over the different graphs.

System	PDB	No. compounds	No. edges	R^2	Edgewise RMSE	Pairwise RMSE
HSP90	3HVD	10	16	0.46	1.47 ± 0.26	2.00 ± 0.16
HSP90	3RKZ	7	9	0.97	0.61 ± 0.10	0.70 ± 0.09
MHT1	5ANT	3	3	1.00	0.70 ± 0.18	0.70 ± 0.18
BACE1	2B8V	2	1	1.00	0.63 ± 0.00	0.62 ± 0.00
BACE1	2Q15	6	9	0.66	0.85 ± 0.29	0.88 ± 0.17
CK2	2PVN	2	1	1.00	1.25 ± 0.00	1.26 ± 0.00
CHK1	2E9P	4	5	0.00	0.95 ± 0.14	1.22 ± 0.26
	Total/weighted mean	34	44	0.66 [0.42, 0.92]	1.09 [0.70, 1.36]	1.32 [0.78, 1.74]

The crystal structure that Yu et al. based their model on (PDB code 3HVA) has ligand 11 bound. Another crystal structure exists with ligand 16 bound (PDB code 3HVD). When this structure was prepared and used in FEP+, the pairwise RMSE reduced to around 2 kcal mol^{-1}, which was due to reducing the error of ligands 16, 17, and 18 by around 0.5 kcal mol^{-1}. The reason for this structural sensitivity of the predictions was not discovered.

The linkers of 17 and 18 were remodeled with Schrödinger's latest macrocycle modeling software. Additionally, to increase the linker sampling in the complex and the solvent, a custom core was created that left all the linkers unmapped and the torsion potentials in the linker were scaled to zero using the FEP+ flag '-modify_dihe'. The overall pairwise error after these adjustments remained at 2.00 kcal mol^{-1}. Longer simulations with more lambda windows did not alter this value by a statistically significant degree.

CK2

This series consists of a single edge between two molecules: one macrocycle (named ligand 15) and one acyclic molecule (named ligand 14). The same ligand binding modes and prepared protein structure as Yu et al. was reused in this study.

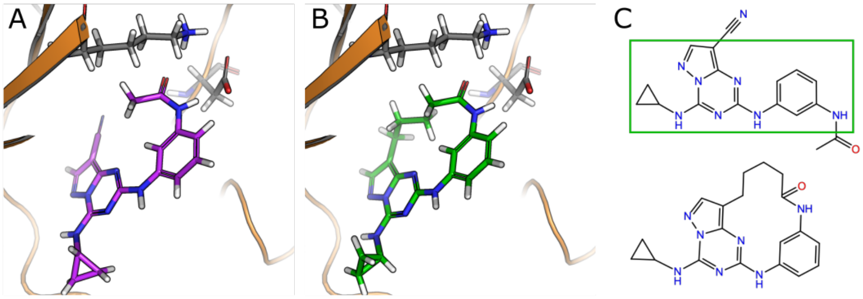

Supplementary Figure 16: The two ligands in the CK2 macrocyclization map. Panel A shows the acyclic ligand and and panel B shows the macrocycle. LYS 68 and ASO 175 are shown with gray carbon atoms. Panel A is taken from the crystal structure (PDB code 2PVN) and it shows the acyclic molecule binding with the amide group in the cis conformation. Panel C shows the 2D image of the the two molecules. The green box indicates which atoms were selected as the custom core. Excluding the amide torsion from the custom core is necessary to account for the strain induced on the acyclic compound when bound to the complex.

Using the automatic atom mapping, the binding free energy of the macrocycle relative to the acyclic molecule was substantially underpredicted, with an error of around 3.4 kcal mol^{-1} compared to the experimental relative binding free energy. The crystal structure of the bound acyclic ligand (PDB code 2PVN and shown in Supplementary Figure 16) indicates that the ligand amide group is in the cis conformation and makes hydrogen bonds with LYS 68 and ASP 175. The amide group was mapped between the two molecules and did not rotate during the FEP simulations. Amide cis conformations are higher in energy than trans conformations and it was hypothesized that the FEP calculations had not fully accounted for the difference in strain energy between the two molecules. To ensure the amide torsion was sampled in a revised FEP+ calculation, a custom core was manually selected that left the amide group unmapped (see panel C of Supplementary Figure 16). Also, the torsion barrier heights between the unmapped atoms was scaled to zero in the

intermediate lambda windows. Additionally, other crystal structures of CK2 (2PVH, 2PVJ, 2PVK, 2PVL, 2PVM, and 2PVN) show that LYS 68 could adapt depending on the ligand. Accordingly, LYS 68 was added to the REST region and applied with the new atom mapping.

Applying the custom core and adding LYS 68 to the REST region greatly improved the accuracy of the predicted relative affinity, bringing to 1.25 kcal mol^{-1} of the experimental value. The most significant driver of accuracy was the choice of custom core that left the amide group unmapped. With the new atom mapping, the amide group relaxed to the trans conformation in solvent, meaning it incurs strain when binding to the protein. In contrast, the macrocycle linker of ligand 15 locks the amide group in the cis conformation in solvent and in the protein, meaning no strain penalty is paid when it binds to the protein.

2.1.9 FEP+ buried water set

This data series consists of FEP maps where buried water is destabilized or displaced and was previously collected by ourselves to validate the integration of grand canonical Monte Carlo (GCMC) sampling of water within FEP+ production simulations[65]. While GCMC significantly reduces FEP’s dependence on the starting distribution of water molecules, these simulations were started with structures where water did not sterically overlap with the ligands.

The scytalone dehydratase data set contains ligands whose pKas are uncertain and we have previously investigated the sensitivity of the predictions with respect to using different pKas[65]. Because of this sensitivity, it is not well suited to validating force fields was excluded from the OPLS4 validation set[66].

Supplementary Table 26: The statistics of the FEP graphs in the FEP+ buried water set. The weighted means have been calculated by weighting edgewise RMSEs by the number of edges in the graph and all other statistics by the number of compounds. Confidence intervals have been calculated by bootstrap sampling over the different graphs.

System	PDB	No. compounds	No. edges	R^2	Edgewise RMSE	Pairwise RMSE
BRD4	5I88	8	11	0.00	2.05 ± 0.24	1.48 ± 0.18
CHK1	2E9V	13	19	0.62	1.04 ± 0.12	1.39 ± 0.10
Thrombin	2ZFF	21	20	0.56	0.94 ± 0.21	1.05 ± 0.05
TAF1(2)	5I1Q	8	11	0.83	0.56 ± 0.06	0.51 ± 0.04
HSP90 , Woodhead et al.	2XJJ	4	5	1.00	0.39 ± 0.07	0.36 ± 0.07
Scytalone dehydratase	3STD	7	12	0.77	1.77 ± 0.27	1.83 ± 0.21
Urokinase	1GJ7	4	6	0.86	0.52 ± 0.11	0.52 ± 0.11
HSP90, Kung et al.	3RLP	11	19	0.69	1.51 ± 0.29	1.70 ± 0.14
	Total/weighted mean	76	103	0.62 [0.45, 0.77]	1.29 [0.93, 1.59]	1.28 [0.98, 1.54]

2.1.10 FEP+ charge change set

The set of FEP maps was taken from a previous study of our that was used to validate transformations that changed the total charge of the ligand[67].

2.1.11 Miscellaneous data sets

The systems in this section do not fit neatly into any other category. The number of ligands in the galactin set is a significant expansion on previous FEP efforts with this series and the BTK and HIV1 protease sets have - to the best of our knowledge - not been used in previous FEP studies. This set also includes the CDK8 set from Koehler et al.[33] that was used to validate the CDK8 set from the Merck data set (section 2.1.1) as well as human FAAH which was originally analyzed with FEP+ by Saha et al.[68].

Galectin-3C

The set of ligands in this congeneric series originates from the PhD thesis of Kristoff Peterson[18]. The series analyzed here primarily explores terminal R-group modifications to a scaffold that binds to the surface of the protein. A subset of eight ligands from this series was used in FEP validation studies by Manzoni et al. and Gapsys et al.[69;70]. In this work, we expand the set of ligands used in previous FEP studies from eight to twenty-six, which were taken from tables 1, 2, and 3 from Peterson’s thesis. Following Manzoni et al., the protein structure used here was taken from PDB ID 5E89 and the modeled binding modes were based on the structures used by Gapsys et al. The protein was prepared with the

Supplementary Table 27: The statistics of the FEP graphs in the FEP+ charge-change set. The weighted means have been calculated by weighting edgewise RMSEs by the number of edges in the graph and all other statistics by the number of compounds. Confidence intervals have been calculated by bootstrap sampling over the different graphs.

System	PDB	No. compounds	No. edges	R^2	Edgewise RMSE	Pairwise RMSE
PTP1B	2QBP	3	6	0.89	1.61 ± 0.36	1.61 ± 0.36
TYK2	4GIH	5	12	0.78	1.09 ± 0.36	1.14 ± 0.21
JAK1	4IVD	6	11	0.73	0.81 ± 0.19	0.89 ± 0.15
CDK2	1OIY	3	6	0.97	0.46 ± 0.13	0.46 ± 0.13
Thrombin	3SI4	6	9	0.94	1.41 ± 0.25	1.45 ± 0.24
DLK	5CEQ	5	9	0.98	0.41 ± 0.14	0.47 ± 0.07
IRAK4	4ZTM	5	14	0.70	1.33 ± 0.29	1.33 ± 0.21
ITK	4PQN	4	8	0.14	1.17 ± 0.21	1.06 ± 0.22
JNK1	2GMX	3	8	0.91	1.01 ± 0.21	1.01 ± 0.21
EPHX2	3KOO	4	8	0.82	1.21 ± 0.23	1.39 ± 0.25
EGFR	3LZB	5	16	0.03	1.59 ± 0.31	1.64 ± 0.25
IRAK4	4ZTN	4	13	0.19	1.21 ± 0.23	1.21 ± 0.23
	Total/weighted mean	53	120	0.67 [0.47, 0.84]	1.20 [1.00, 1.36]	1.20 [1.00, 1.37]

Supplementary Table 28: The statistics associated with the FEP maps in the miscellaneous set. The weighted means have been calculated by weighting edgewise RMSEs by the number of edges in the graph and all other statistics by the number of compounds. Confidence intervals have been calculated by bootstrap sampling over the different graphs.

System	PDB	No. compounds	No. edges	R^2	Edgewise RMSE	Pairwise RMSE
CDK8	5CEI	10	16	0.44	1.23 ± 0.41	1.34 ± 0.13
BTK	4ZLZ	6	9	0.78	1.20 ± 0.20	1.21 ± 0.16
Galectin	5E89	26	41	0.76	0.64 ± 0.07	0.60 ± 0.02
HIV1 protease	2CEN	13	19	0.00	1.09 ± 0.19	1.12 ± 0.07
FAAH	6MRG	24	35	0.72	0.99 ± 0.09	1.08 ± 0.04
	Total/weighted mean	79	120	0.59 [0.26, 0.75]	0.96 [0.77, 1.18]	1.01 [0.77, 1.25]

protein preparation wizard with all crystallographic waters retained. Initially, alternate rotamers of the ligands in Table 1 of Peterson's thesis were added to the perturbation map. However, these rotamers were found to readily interconvert during the FEP+ simulations, violating the assumption that is required to apply our simple binding mode correction. Instead, these alternate rotamers were removed for the final accuracy assessment. The final pairwise RMSE for this data set was 0.60 ± 0.02 kcal mol^{-1}.

BTK

The series by Smith et al. consists of different single and double conjugated rings that are substituted onto an aminocinnoline core[71]. The aminocinnoline core is also a ligand in this series. By inspection of two crystal structures from the study (PDB codes 4ZLZ and 4Z3V), the modification of a methylpyridine to an indazole appears to displace a buried water molecule. The structure that includes the buried water molecule (4ZLZ) was prepared using the protein preparation workflow and all crystallographic water molecules were retained. Two ligands with an indazole substituent (ligands 10 and 11) make two hydrogen bonds with the backbone of PHE 413 and GLY 414. The orientation of the indazole is well supported by the electron density of 4Z3V. In the place of the indazole group, Ligand 9 contains an indole group that can be modeled either forming a hydrogen bond with GLY 414 (when in the same orientation as as the indazole substituent) or flipped 180 degrees to form a hydrogen bond with the sidechain carboxamide of ASN 526. Both orientations were included in the perturbation map. The binding mode and orientation of the methylpyridine substituent of ligand 8 is clearly resolved in the electron density of crystal structure 4ZLZ. An alternate orientation of the methylpyridine - flipped 180 degrees relative to the crystallographic pose - was also included in the perturbation map as it also appeared to fit well in the binding pocket.

Interestingly, the extra rotamer state of ligand 8 was predicted to be approximately equally probable as the crystallographic rotamer. Also, the rotamer of ligand 9 that forms a hydrogen bond with ASN 526 was found to be roughly 2 kcal mol^{-1} more favorable than when in the same orientation as the crystallographically resolved indazole group.

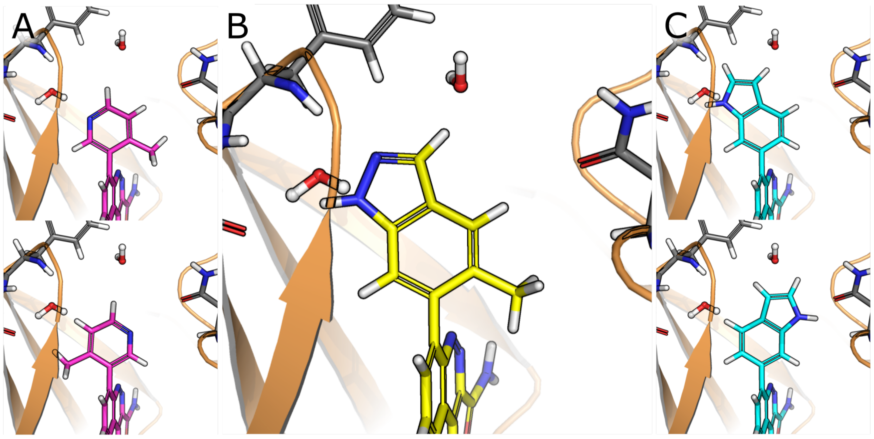

Supplementary Figure 17: Example ligands and binding modes from the BTK series from Smith et al. The top of panel A shows the crystallographic binding mode of ligand 8 (PDB code 4ZLZ). A water molecule can be seen bridging the interaction between the protein and the ligand. The bottom of panel A shows an alternate rotamer of ligand 8 that was also included. Panel C shows ligand 11 in the binding mode as seen in the crystal structure 4Z3V. A buried water molecule appears to be displaced relative to ligand 8. Panel C shows the two orientations of the indole group of ligand 9 that were included in the perturbation map. The most favorable orientation is shown in the lower half of the panel.

When these additional rotamer states of ligand 8 and 9 were ignored in the accuracy calculation, the pairwise RMSE was 1.47 ± 0.17 kcal mol^{-1}. When the results were processed with the binding mode correction protocol, the pairwise RMSE decreased to 1.21 ± 0.16 kcal mol^{-1}.

HIV1 protease

This series was taken from Table 1 from Ekegren et al. and consists of thirteen molecules with different solvent facing substituents[72]. To our knowledge, this is the first time this data set has been used in an FEP study. One of the crystal structures associated with the study (PDB code 2CEN) was downloaded and prepared using the standard workflow. All ligands substituents were modeled into the binding site using the MCS docking workflow using ligand 11 (the crystallographic ligand) as a reference. Using FEP+, the pairwise RMSE for this data set was found to be 1.12 ± 0.07 kcal mol^{-1}.

FAAH

This data set follows the work of Saha et al.[68] who originally applied FEP+ to the this system. The binding affinities of these ligands where measured using human FAAH, but the closest X-ray crystal structures for this protein are humanized rat FAAH, where key residues in the binding site have been mutated to their human counterparts. We are grateful to Mark Seierstad for informing us that unpublished mutagenesis studies support the expectation that IC50s using this humanized rat FAAH are close to human FAAH.

The ligands in this series have a central chiral center and the study of Saha et al. used a number of ligands whose experimental affinities were from a racemic mixture. As FEP requires the chiralty of the simulated ligands to match the chirality of the measured ligands, the ligands with racemic binding affinities were excluded from this study.

The protein structure was prepared from PDB entry 6MRG and ligands were docked using the crystallographic ligand as a reference. The majority of the FAAH ligands used in this benchmark consist of modifications on a terminal aryl group. As noted by Saha et al., the orientation of some of these groups is uncertain. As a result, the entire aryl group was added to the REST region to facilitate rotamer sampling.

2.1.12 FEP+ R-group set

The data set originally assembled by ourselves[73], sometimes referred to as the "JACS set", has become a standard benchmark for the development of FEP methods so extra attention was paid this systems to ensure that the protein and ligand structures are the most appropriate. This section outlines the additional validation that we performed on the PTB1B, JNK1, and Thrombin systems.

Supplementary Table 29: The statistics of the FEP+ R-group set. The weighted means have been calculated by weighting edgewise RMSEs by the number of edges in the graph and all other statistics by the number of compounds. Confidence intervals have been calculated by bootstrap sampling over the different graphs.

System	PDB	No. compounds	No. edges	R^2	Edgewise RMSE	Pairwise RMSE
Mcl1	4HW3	42	123	0.49	1.17 ± 0.08	1.24 ± 0.03
CDK2	1H1Q	16	25	0.42	1.16 ± 0.11	1.31 ± 0.07
P38	3FLN	34	56	0.49	0.89 ± 0.09	1.16 ± 0.03
TYK2	4GIH	16	24	0.86	0.82 ± 0.12	0.69 ± 0.04
BACE1	4DJW	36	58	0.39	1.04 ± 0.09	1.08 ± 0.03
JNK1	2GMX	21	34	0.71	0.65 ± 0.07	0.83 ± 0.04
Thrombin	2ZFF	11	14	0.68	0.94 ± 0.17	0.79 ± 0.07
PTP1B	2QBS	23	49	0.85	0.64 ± 0.05	0.74 ± 0.03
	Total/weighted mean	199	383	0.57 [0.47, 0.71]	0.98 [0.79, 1.10]	1.05 [0.88, 1.16]

PTP1B

All ligands in this ligand series have two adjacent carboxylic acid R-groups that bind to a positively charged, shallow pocket on the surface of PTB1B. Within this shallow pocket is CYS 215, whose side chain is buried amongst backbone amines (see Supplementary Figure 18). In our previous publications that use this system and series, CYS 215 was treated as being deprotonated. However, this choice was recently questioned by Gapsys et al. who found that PROPKA predicted this residue to be protonated and who observed improved FEP accuracy with this setting[70]. To see which protonation state is consistent with our forcefield and sampling methodology, we calculated the pKa or CYS 215 when PTB1B was complexed with four representative ligands from the series using our protein mutation FEP workflow. The ligands, free energy differences, and calculated pKas are shown in Supplementary Table 30. The four calculated pKas are consistent with CYS 215 being deprotonated. This protonation state is expected to apply to all ligands in the series given the high level of similarity of the ligands in the vicinity of CYS 215.

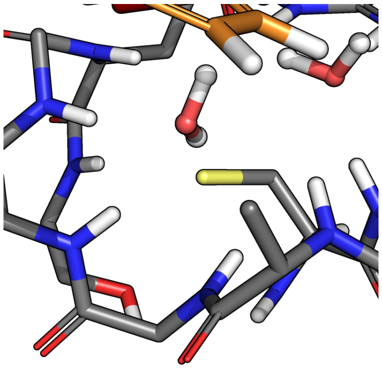

Supplementary Figure 18: The deprotonated form of CYS 215 in PTB1B (PDB code 2QBS). The protein is shown with gray carbon atoms and the ligand (in the upper part of the figure) is shown with orange carbon atoms. This side chain has previously been treated as deprotonated in our FEP studies. We verififed this choice by calculating the pKa of CYS with our protein mutation FEP workflow.

JNK1

The ligands in this series primarily explore R-group modifications to an aryl group in a solvent exposed region. Four ligands in the series have aryl groups that are symmetric with respect to 180 degree torsion rotations, whereas the rest (seventeen in total) have two torsion rotations that are sterically allowed. As only one orientation of these groups were included in the original perturbation map, all non symmetric aryl groups had additional rotamers added. This increased the number of nodes in the map from 21 to 38. Supplementary Figure 19 shows some examples of the ligands and the additional, alternate rotations.

Supplementary Table 30: The calculated relative free energy to deprotonate CYS 215 for four ligands bound to PTB1B. The reference pKa for CYS that was used in this calculations was 8.2. These results support the use of the deprotonated form of CYS (CYM) in the ligands FEP calculations.

Ligand name	Deprotonation $\Delta\Delta G$ (kcal mol^{-1})	pKa in complex
20667	-9.98	0.93
23477	-9.99	0.92
23479	-9.42	1.34
23486	-8.98	1.66

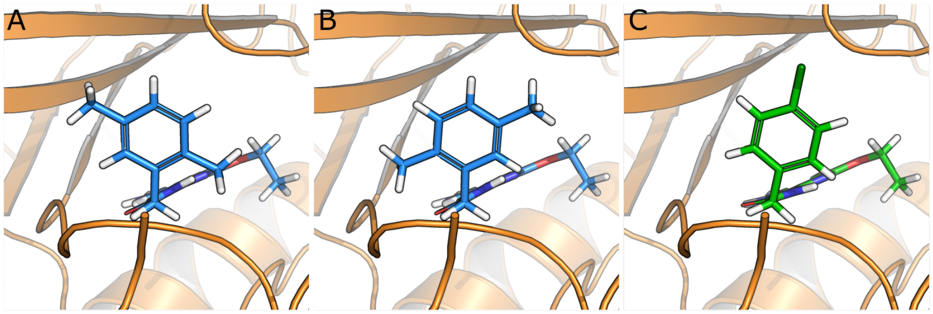

Supplementary Figure 19: Two example ligands from the JNK1 series. Additional rotameric states were added to the perturbation map for all non symmetric solvent exposed aryl groups. Panel A shows the original orientation of the dimethylphenyl group in ligand 18635 and panel B shows the additional, flipped torsion that was added to the map. The additional orientation (panel B) was found to be only 0.13 kcal mol^{-1} less favorable than the original orientation (panel A), making both orientations almost equiprobable. Panel C shows ligand 18627, which has a symmetric aryl group (chlorophenyl) that required a symmetry correction.

All original aryl orientations were found to be more favorable than the additional poses, although five of the additional poses were found to be within 1 kcal mol^{-1} of the original, such as ligand 18635 shown in Supplementary Figure 19. The symmetric aryl groups, such as ligand 18627 in Supplementary Figure 19, were not found to fully rotate when in the complex but could in solvent, necessitating a symmetry correction to the four ligands. When the completed map was processed by ignoring the additional poses, the pairwise RMSE was 1.14 ± 0.05 kcal mol^{-1}. Applying the binding mode correction reduced the pairwise RMSE to 0.94 ± 0.04 kcal mol^{-1}, which was further reduced to 0.83 ± 0.04 kcal mol^{-1} after applying the symmetry correction to four ligands with symmetric aryl groups. This final RMSE is used in the benchmark assessment.

We note that the crystal structure used as the basis of the protein model has resolution of 3.5 Å that is higher than most other crystal structures that are used in this benchmark. The poorer quality of this crystal structure was highlighted by Hahn et al., who suggested alternative crystal structures. Future developments of this system will explore alternate, higher quality crystal structures.

MCL1

As the protein structure originally used for this map had the crystallographic waters removed, the protein was downloaded from the PDB (code 4HW3) and reprepared to retain all water molecules. The map comprises many R-group modifications that can be positioned either in a small hydrophobic pocket or towards solvent. In reanalyzing the ligand poses, some inconsistencies were found between the ligands, such as one ligand having an R-group pointed to the hydrophobic pocket and another, with the same R-group, pointing towards solvent (see Supplementary Figure 20). To remove such inconsistencies, additional rotamers were added to the perturbation map for all ligands that did not have symmetric R-groups. Out of 42 ligands, 26 ligands had additional rotamers added to the map. Eight of the rotamers were found to be more favorable than the original poses, with many having near equiprobable orientations. All additional rotamers were post-processed with the binding mode correction.

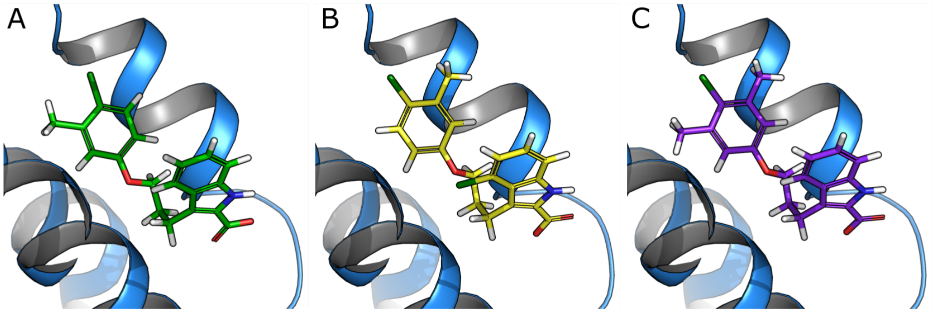

Supplementary Figure 20: Representative ligands from the 2015 MCL1 data set. Ligand 35 (panel A) and ligand 49 (panel B) differ only by a Cl in the core, but the methyl decoration in the chlorobenzene group was previously modeled in different orientations. As the methyl can point either towards the protein pocket (like in panel A) or towards solvent (like in panel B), both orientations were included for ligand 35 and 49 in the current perturbation map. Panel C shows ligand 37 which has a dimethylated chlorobenzene that is symmetric with respect to 180 degree rotations.

There are eighteen ligands in the map that have R-groups with two degrees of rotational symmetry which were considered for the symmetry correction. It was found that, in the complex, these symmetrical groups either sampled both rotamers equally well, or at worst, inconsistently. As a result, no symmetry corrections were applied to this map.

The pairwise RMSE was 1.41 ± 0.03 kcal mol^{-1} on the original MCL1 perturbation map, which reduced to 1.24 ± 0.03 kcal mol^{-1} after updating the protein structure and adding the additional rotamers.

Thrombin

The eleven ligands in this series have binding affinities that have been measured with ITC and are a subset of the ligands that are present in the thrombin map in the buried water set (Section 2.1.9. In the larger thrombin map, which contains 21 ligands, the affinities were measured using a competitive inhibition assay. Each ligand explores R-group modifications to a terminal phenyl group. The undecorated phenyl group (ligand 5) is also part of this subset. For all non-symmetric ligands, the buried water set map used two rotamer states that were processed with the binding mode correction protocol[65], and the lack of symmetric rotamer sampling of ligand 5 in the complex required a symmetry correction. In this ITC measured subset, the largest non symmetric ligand is 2,5 dichlorophenyl, so it was decided to leave these aryl groups unmapped in the FEP rather than to add additional rotamers. It was expected that the different orientations of these small groups would be sufficiently sampled with our (automatically applied) replica exchange solute tempering method. Indeed, after completion of the simulations, the rotationally symmetric phenyl group of ligand 5 was found to fully sample both orientations in the complex, suggesting that the other groups were appropriately sampled. The pairwise RMSE for this map was 0.79 ± 0.07 kcal mol^{-1}.

As the same ligands in this series appear in the larger thrombin map that is used to assess FEP+ accuracy for water displacement, the weighting of the pairwise RMSEs in overall assessment was adjusted. In this benchmark exercise, the overall pairwise RMSE is calculated by weighting the pairwise RMSE of each map by the number of ligands in the data set. Instead, the pairwise RMSE of the larger thrombin map was weighted by the number of ligands that are in that map and *not* in this subset (a total of ten), whereas the pairwise RMSE of this subset were weighted by the number of ligands in the map (eleven). This ensures that the same ligands are not double counted in the final weighted average.

2.1.13 OPLS stress set

This set was constructed for validating updates to the OPLS forcefield[66;74] and primarily consists of heterocycle modifications. All protein and ligand structures were taken directly from the OPLS4 manuscript[66] with the exception of the FXa Yoshikawa et al. set, which is detailed below.

FXa set 1 and set 2

The original OPLS stress set included factor Xa ligands from two papers by Yoshikawa et al.[75;76]. As these two sets of ligands share a common core and were assayed with the same biochemical assay, it was decided to merge these sets into a single map. Previously, the first and second set of factor Xa ligands contained 10 and 8 ligands. Additional ligands from the YoshiKawa investigations were also added to this assessment which brought the total number of ligands to 27.

Supplementary Table 31: The statistics of the OPLS stress set. The weighted means have been calculated by weighting edgewise RMSEs by the number of edges in the graph and all other statistics by the number of compounds. Confidence intervals have been calculated by bootstrap sampling over the different graphs.

System	PDB	No. compounds	No. edges	R^2	Edgewise RMSE	Pairwise RMSE
BACE1 cr1	4DJW	11	13	0.01	1.49 ± 0.20	1.72 ± 0.15
BACE1 cr2	4DJW	12	17	0.61	1.15 ± 0.17	1.27 ± 0.09
BACE1 hc1	3NSH	7	9	0.67	1.07 ± 0.23	0.99 ± 0.14
BACE1 hc2	3NSH	10	13	0.01	1.42 ± 0.31	1.55 ± 0.14
BACE1 pb	3NSH	4	6	0.80	0.61 ± 0.12	0.61 ± 0.12
CHK1 set 1	2AYP	3	3	0.41	1.81 ± 0.48	1.81 ± 0.48
CHK1 set 2	2HY0	2	1	1.00	0.11 ± 0.00	0.11 ± 0.00
CHK1 set 3	2YDK	4	6	0.93	0.57 ± 0.12	0.57 ± 0.12
CHK1 set 4	2YDJ	2	1	1.00	0.67 ± 0.00	0.67 ± 0.00
CHK1 set 5	3PA3	3	3	0.94	1.01 ± 0.19	1.01 ± 0.19
CHK1 set 6	3U9N	3	3	0.91	1.26 ± 0.38	1.26 ± 0.38
CHK1 set 7	2E9V	3	3	0.68	1.31 ± 0.28	1.31 ± 0.28
FXa Yoshikawa et al.	3IIT	27	46	0.56	1.88 ± 0.19	1.83 ± 0.06
FXa set 3	2VVV	4	6	0.18	0.68 ± 0.10	0.68 ± 0.10
FXa set 4	2J95	11	14	0.42	1.20 ± 0.20	1.42 ± 0.11
FXa set 5	3IIT	2	1	1.00	0.67 ± 0.00	0.68 ± 0.00
FXa set 6	2J95	6	13	0.82	1.36 ± 0.19	1.42 ± 0.18
	Total/weighted mean	114	158	0.52 [0.35, 0.68]	1.44 [1.11, 1.66]	1.44 [1.13, 1.63]

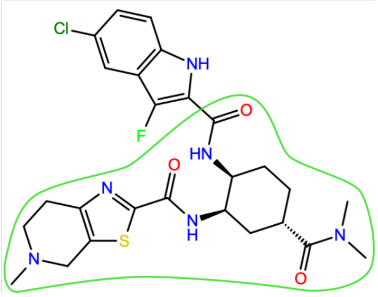

Supplementary Figure 21: An example ligand from the series of ligands by Yoshikawa et al., which is referred to as 8j in the original publication. The atoms within the curved green line were included in an FEP custom core; the fused ring outside this area was decoupled in the intermediate lambda windows. In this ligand, the dihedral angle between this fused ring and the core sampled 180 degree rotations when bound to the protein.

The ligands in these series differ in a chlorinated fused ring, whose orientation in the crystallized protein-ligand structures is unequivocal. Nevertheless, it may be that perturbations to this fused cause this ring to rotate when bound to the protein. To account for this possibility, a custom core was created (see Supplementary Figure 21) that decoupled the fused ring in the intermediate lambda windows. In the intermediate lambda windows, the torsion potentials to this fused ring were also scaled to zero with the '-modify-dihe' option in FEP+. In the simulations, 180 degree rotations of the fused ring were observed with some ligands, such as ligand 8j in Supplementary Figure 21.

2.1.14 OPLS drug discovery

This set of maps has also been used to validate the OPLS forcefield[66;74] and comprises of structures and ligands from drug discovery projects that cannot be shared.

2.2 On the variance of accuracy from different FEP+ approaches

We have used prior FEP+ predictions on the R-group set to estimate the variability that arises from changing the FEP methodology. This data set has been a standard benchmark for FEP+ since 2015, and comprises 199 ligands across 8 targets. While there have been minor adjustments to the structures since 2015, it has remained a very stable data

Supplementary Table 32: The statistics of the OPLS drug discovery set. The weighted means have been calculated by weighting edgewise RMSEs by the number of edges in the graph and all other statistics by the number of compounds. Confidence intervals have been calculated by bootstrap sampling over the different graphs.

System	No. compounds	No. edges	R^2	Edgewise RMSE	Pairwise RMSE
A 0	2	1	1.00	0.08 ± 0.00	0.08 ± 0.00
A 1	7	8	0.83	0.70 ± 0.11	0.85 ± 0.11
A 3	2	1	1.00	0.26 ± 0.00	0.26 ± 0.00
A 5	2	1	1.00	0.07 ± 0.00	0.07 ± 0.00
B	4	5	0.73	0.70 ± 0.15	0.66 ± 0.14
C 1	10	14	0.74	1.25 ± 0.14	1.18 ± 0.10
C 2	6	8	0.85	0.65 ± 0.11	0.77 ± 0.11
C 3	5	6	0.96	1.07 ± 0.20	1.01 ± 0.14
D	10	16	0.49	1.30 ± 0.15	1.82 ± 0.15
E 1	16	22	0.67	0.80 ± 0.14	0.99 ± 0.07
E 2	12	16	0.26	1.21 ± 0.17	1.50 ± 0.12
E 3	17	24	0.32	1.06 ± 0.12	1.06 ± 0.05
Total/weighted mean	93	122	0.60 [0.47, 0.79]	1.03 [0.84, 1.16]	1.16 [0.90, 1.38]

set that has been widely used in the community. The FEP results from this study will not be used in this comparison because, as discussed in section 2.1.12, we made several significant improvements to the JNK1, MCL1, and thrombin data sets. Instead, we use the results from the original 2015 publication and 3 subsequent force field development papers (up to 2021). The sampling methodology and force field underwent significant improvements between 2015 and 2021, such as enhanced water sampling and improved charged interactions. The publications from 2015 to 2021 also used different amounts of sampling time. The data shown in Supplementary Table 33 demonstrates that different computational approaches, even those that are closely related, can have variable accuracy. One can use the range of the metrics shown (such as RMSE) as an approximate estimate of this variability.

Supplementary Table 33: The weighted pairwise RMSEs, MUEs (in kcal mol^{-1}), R^2 and Kendall τ of each method on the FEP+ R-group set (colloquially known as the "JACS" set). The errors reflect the standard error after bootstrapping over every ligand pair within each target for the pairwise RMSE and bootstrapping over the ligands in each series for the weighted R-squared and Kendall's τ. On these data, the null model, in which all $\Delta\Delta G$s have a predicted free energy of zero, has a weighted RMSE and MUE of 1.46 kcal mol^{-1} and 1.16 kcal mol^{-1}, respectively, and a R-squared and Kendall's τ equal to zero.

Force field and data source	Time per λ (ns)	Weighted pairwise RMSE	Weighted pairwise MUE	Weighted R^2	Weighted Kendall's τ
OPLS2.1[73]	5	1.34 ± 0.02	1.06 ± 0.02	0.57 ± 0.04	0.57 ± 0.04
OPLS3[74]	25	1.20 ± 0.02	0.94 ± 0.01	0.51 ± 0.04	0.50 ± 0.04
OPLS3e[74]	25	1.09 ± 0.01	0.84 ± 0.01	0.62 ± 0.04	0.59 ± 0.04
OPLS4[66]	20	1.09 ± 0.02	0.86 ± 0.01	0.61 ± 0.04	0.59 ± 0.04

As seen in Supplementary Table 33 OPLS4 and OPLS3e appear to have a similar level of accuracy on the R-group set, most notably with the weighted pairwise RMSE. To demonstrate the ability for larger, more heterogeneous data sets to discriminate between methods, we reanalyzed the performance of OPLS3e and OPLS4 force fields on the OPLS4 validation data set using the metrics used in this work. This validation set contains a total of 512 protein-ligand pairs. As above, the raw predictions were taken from the original publications as opposed to from this work (due to the modifications made to the data sets) and were re-analysed using the same metrics as used here.

The results in Supplementary Table 34 shows a clear difference in accuracy between OPLS3e and OPLS4. As the FEP benchmark set is even larger and more heterogeneous than the OPLS validation set, we expect that the variation of the accuracy from different computational approaches to be even more apparent.

Supplementary Table 34: A comparison of the accuracy of OPLS3e and OPLS4 on the OPLS4 validation set[66]. Unlike the 2015 data set, the expanded data set is more able to distinguish between the improved accuracy of OPLS4 compared to OPLS3e. The RMSEs are in kcal mol^{-1}.

	OPLS3e	OPLS4
Weighted pairwise RMSE	1.34 ± 0.02	1.17 ± 0.02
Weighted R^2	0.57 ± 0.03	0.60 ± 0.03
Weighted Kendall's τ	0.52 ± 0.03	0.54 ± 0.03

Supplementary References

[1] Julie Qi Hang, Yanli Yang, Seth F. Harris, Vincent Leveque, Hannah J. Whittington, Sonal Rajyaguru, Gloria Ao-Leong, Matthew F. McCown, April Wong, Anthony M. Giannetti, Sophie Le Pogam, Francisco Talamás, Nick Cammack, Isabel Nájera, and Klaus Klumpp. Slow binding inhibition and mechanism of resistance of non-nucleoside polymerase inhibitors of Hepatitis C virus. *J. Biol. Chem.*, 284(23):15517–15529, 2009. ISSN 00219258. doi:10.1074/jbc.M808889200.

[2] Christina E.M. Schindler, Hannah Baumann, Andreas Blum, Dietrich Böse, Hans Peter Buchstaller, Lars Burgdorf, Daniel Cappel, Eugene Chekler, Paul Czodrowski, Dieter Dorsch, Merveille K.I. Eguida, Bruce Follows, Thomas Fuchß, Ulrich Grädler, Jakub Gunera, Theresa Johnson, Catherine Jorand Lebrun, Srinivasa Karra, Markus Klein, Tim Knehans, Lisa Koetzner, Mireille Krier, Matthias Leiendecker, Birgitta Leuthner, Liwei Li, Igor Mochalkin, Djordje Musil, Constantin Neagu, Friedrich Rippmann, Kai Schiemann, Robert Schulz, Thomas Steinbrecher, Eva Maria Tanzer, Andrea Unzue Lopez, Ariele Viacava Follis, Ansgar Wegener, and Daniel Kuhn. Large-scale assessment of binding free energy calculations in active drug discovery projects. *J. Chem. Inf. Model.*, 60(11): 5457–5474, 2020. ISSN 15205142. doi:10.1021/acs.jcim.0c00900.

[3] D. G. Myszka, Y. N. Abdiche, F. Arisaka, O. Byron, E. Eisenstein, P. Hensley, J. A. Thomson, C. R. Lombardo, F. Schwarz, W. Stafford, and M. L. Doyle. The ABRF-MIRG'02 study: Assembly state, thermodynamic, and kinetic analysis of an enzyme/inhibitor interaction. *J. Biomol. Tech.*, 14(4):247–269, 2003. ISSN 15240215.

[4] Michelle J. Cannon, Giuseppe A. Papalia, Iva Navratilova, Robert J. Fisher, Lindsey R. Roberts, Karen M. Worthy, Andrew G. Stephen, Gerardo R. Marchesini, Edward J. Collins, Dave Casper, Huawei Qiu, Daulet Satpaev, Stefano F. Liparoto, Dax A. Rice, Inna I. Gorshkova, Ryan J. Darling, Donald B. Bennett, Michael Sekar, Eric Hommema, Amy M. Liang, Eric S. Day, Jean Inman, Shannon M. Karlicek, Stephen J. Ullrich, Dianne Hodges, Teresa Chu, Eric Sullivan, Jack Simpson, Ashique Rafique, Béatrice Luginbühl, Susanne Nyholm Westin, Magdalena Bynum, Paul Cachia, Yue Jin Li, Daniel Kao, Amy Neurauter, Melanie Wong, Michael Swanson, and David G. Myszka. Comparative analyses of a small molecule/enzyme interaction by multiple users of Biacore technology. *Anal. Biochem.*, 330(1):98–113, 2004. ISSN 00032697. doi:10.1016/j.ab.2004.02.027.

[5] Giuseppe A. Papalia, Stephanie Leavitt, Maggie A. Bynum, Phinikoula S. Katsamba, Rosemarie Wilton, Huawei Qiu, Mieke Steukers, Siming Wang, Lakshman Bindu, Sanjay Phogat, Anthony M. Giannetti, Thomas E. Ryan, Victoria A. Pudlak, Katarzyna Matusiewicz, Klaus M. Michelson, Agnes Nowakowski, Anh Pham-Baginski, Jonathan Brooks, Bryan C. Tieman, Barry D. Bruce, Michael Vaughn, Michael Baksh, Yun Hee Cho, Mieke De Wit, Alexandra Smets, Johan Vandersmissen, Lieve Michiels, and David G. Myszka. Comparative analysis of 10 small molecules binding to carbonic anhydrase II by different investigators using Biacore technology. *Anal. Biochem.*, 359(1):94–105, 2006. ISSN 10960309. doi:10.1016/j.ab.2006.08.021.

[6] Matthias C Jecklin, Stefan Schauer, Christoph E Dumelin, and Renato Zenobi. Label-free determination of protein-ligand binding constants using mass spectrometry and validation using surface plasmon resonance and isothermal titration calorimetry. *J. Mol. Recognit.*, 22(4):319–329, 2009. ISSN 1099-1352. doi:10.1002/jmr.951. URL `http://dx.doi.org/10.1002/jmr.951`.

[7] Nicolas Bocquet, Josiane Kohler, Melanie N. Hug, Eric A. Kusznir, Arne C. Rufer, Roger J. Dawson, Michael Hennig, Armin Ruf, Walter Huber, and Sylwia Huber. Real-time monitoring of binding events on a thermostabilized human A2A receptor embedded in a lipid bilayer by surface plasmon resonance. *Biochim. Biophys. Acta - Biomembr.*, 1848(5):1224–1233, 2015. ISSN 18792642. doi:10.1016/j.bbamem.2015.02.014. URL `http://dx.doi.org/10.1016/j.bbamem.2015.02.014`.

[8] Peter D. Ycas, Huda Zahid, Alice Chan, Noelle M. Olson, Jorden A. Johnson, Siva K. Talluri, Ernst Schonbrunn, and William C.K. Pomerantz. New inhibitors for the BPTF bromodomain enabled by structural biology and biophysical assay development. *Org. Biomol. Chem.*, 18(27):5174–5182, 2020. ISSN 14770520. doi:10.1039/d0ob00506a.

[9] Eliane Mandine, Dominique Gofflo, Vronique Jean-Baptiste, Edoardo Sarubbi, Gaetan Touyer, Pierre Deprez, and Dominique Lesuisse. Src homology-2 domain binding assays by scintillation proximity and surface plasmon resonance. *J. Mol. Recognit.*, 14(4):254–260, 2001. ISSN 09523499. doi:10.1002/jmr.542.

[10] Steven M. Murphy, Marc Palmer, Michelle Fontilla Poole, Linas Padegimas, Karen Hunady, Joel Danzig, Sikander Gill, Rajwant Gill, Anthony Ting, Bruce Sherf, Kurt Brunden, and Alain Stricker-Krongrad. Evaluation of functional and binding assays in cells expressing either recombinant or endogenous hERG channel. *J. Pharmacol. Toxicol. Methods*, 54(1):42–55, 2006. ISSN 10568719. doi:10.1016/j.vascn.2005.10.003.

[11] Iva Navratilova, Giuseppe A. Papalia, Rebecca L. Rich, Daniel Bedinger, Susan Brophy, Brad Condon, Ta Deng, Anne W. Emerick, Hann Wen Guan, Tanya Hayden, Thomas Heutmekers, Bart Hoorelbeke, Mark C. McCroskey, Mary M. Murphy, Terry Nakagawa, Fabio Parmeggiani, Xiaochun Qin, Sabina Rebe, Nenad Tomasevic,

Tiffany Tsang, M. Brett Waddell, Fred Feiyu Zhang, Stephanie Leavitt, and David G. Myszka. Thermodynamic benchmark study using Biacore technology. *Anal. Biochem.*, 364(1):67–77, 2007. ISSN 10960309. doi:10.1016/j.ab.2007.01.031.

[12] Bandaru Narasimha Murthy, Sharmistha Sinha, Avadhesha Surolia, Shantinath S. Indi, and Narayanaswamy Jayaraman. SPR and ITC determination of the kinetics and the thermodynamics of bivalent versus monovalent sugar ligand-lectin interactions. *Glycoconj. J.*, 25(4):313–321, 2008. ISSN 02820080. doi:10.1007/s10719-007-9076-6.

[13] JA Aaron. Structure, function and applications of metal-requiring enzymes: Carbonic anhydrase and epi-isozizaene synthase. *Publicly Access. Penn Diss.*, page 171, 2010. URL `http://repository.upenn.edu/cgi/viewcontent.cgi?article=1156{&}context=edissertations`.

[14] Jennifer L. Mason, Chrysanthe Spais, Jean Husten, Eric Prouty, Mark S. Albom, Sheryl L. Meyer, Mark A. Ator, and Thelma S. Angeles. Comparison of lanthascreen Eu kinase binding assay and surface plasmon resonance method in elucidating the binding kinetics of focal adhesion kinase inhibitors. *Assay Drug Dev. Technol.*, 10(5): 468–475, 2012. ISSN 1540658X. doi:10.1089/adt.2012.453.

[15] Janet Newman, Olan Dolezal, Vincent Fazio, Tom Caradoc-Davies, and Thomas S. Peat. The DINGO dataset: A comprehensive set of data for the SAMPL challenge. *J. Comput. Aided. Mol. Des.*, 26(5):497–503, 2012. ISSN 0920654X. doi:10.1007/s10822-011-9521-2.

[16] Tiphaine Rogez-Florent, Laetitia Duhamel, Laurence Goossens, Perrine Six, Anne Sophie Drucbert, Patrick Depreux, Pierre Marie Danzé, David Landy, Jean François Goossens, and Catherine Foulon. Label-free characterization of carbonic anhydrase - Novel inhibitor interactions using surface plasmon resonance, isothermal titration calorimetry and fluorescence-based thermal shift assays. *J. Mol. Recognit.*, 27(1):46–56, 2014. ISSN 09523499. doi:10.1002/jmr.2330.

[17] Gisela Schnapp, Thomas Klein, Yvette Hoevels, Remko A. Bakker, and Herbert Nar. Comparative analysis of binding kinetics and thermodynamics of dipeptidyl peptidase-4 inhibitors and their relationship to structure. *J. Med. Chem.*, 59(16):7466–7477, 2016. ISSN 15204804. doi:10.1021/acs.jmedchem.6b00475.

[18] Kristoffer Peterson. *Molecular basis for galectin-ligand interactions. Design , synthesis and analysis*. PhD thesis, Lund University, 2018.

[19] Zaneta Nikolovska-Coleska, Renxiao Wang, Xueliang Fang, Hongguang Pan, York Tomita, Peng Li, Peter P. Roller, Krzysztof Krajewski, Naoyuki G. Saito, Jeanne A. Stuckey, and Shaomeng Wang. Development and optimization of a binding assay for the XIAP BIR3 domain using fluorescence polarization. *Anal. Biochem.*, 332 (2):261–273, 2004. ISSN 00032697. doi:10.1016/j.ab.2004.05.055.

[20] Bernhard Baum, Menshawy Mohamed, Mohamed Zayed, Christof Gerlach, Andreas Heine, David Hangauer, and Gerhard Klebe. More than a Simple Lipophilic Contact: A Detailed Thermodynamic Analysis of Nonbasic Residues in the S1 Pocket of Thrombin. *J. Mol. Biol.*, 390(1):56–69, 2009. ISSN 00222836. doi:10.1016/j.jmb.2009.04.051. URL `http://dx.doi.org/10.1016/j.jmb.2009.04.051`.

[21] Pei Pei Kung, Piet Jan Sinnema, Paul Richardson, Michael J. Hickey, Ketan S. Gajiwala, Fen Wang, Buwen Huang, Guy McClellan, Jeff Wang, Karen Maegley, Simon Bergqvist, Pramod P. Mehta, and Robert Kania. Design strategies to target crystallographic waters applied to the Hsp90 molecular chaperone. *Bioorganic Med. Chem. Lett.*, 21(12):3557–3562, 2011. ISSN 0960894X. doi:10.1016/j.bmcl.2011.04.130. URL `http://dx.doi.org/10.1016/j.bmcl.2011.04.130`.

[22] Shiliang Li, Hongling Xu, Shichao Cui, Fangshu Wu, Youli Zhang, Ming-bo Su, Yinghui Gong, Shaobing Qiu, Qian Jiao, Chun Qin, Jiwei Shan, Ming Zhang, Jiawei Wang, Qiao Yin, Minghao Xu, Xiaofeng Liu, Rui Wang, Lili Zhu, Jia Li, Yufang Xu, Hualiang Jiang, Zhenjiang Zhao, Jingya Li, and Honglin Li. Discovery and Rational Design of Natural-Product-Derived 2-Phenyl-3,4-dihydro-2H-benzo[f]chromen-3-amine Analogs as Novel and Potent Dipeptidyl Peptidase 4 (DPP-4) Inhibitors for the Treatment of Type 2 Diabetes. *J. Med. Chem.*, 59(14): 6772–6790, 2016. ISSN 15204804. doi:10.1021/acs.jmedchem.6b00505.

[23] Terry D. Crawford, Vickie Tsui, E. Megan Flynn, Shumei Wang, Alexander M. Taylor, Alexandre Côté, James E. Audia, Maureen H. Beresini, Daniel J. Burdick, Richard Cummings, Les A. Dakin, Martin Duplessis, Andrew C. Good, Michael C. Hewitt, Hon Ren Huang, Hariharan Jayaram, James R. Kiefer, Ying Jiang, Jeremy Murray, Christopher G. Nasveschuk, Eneida Pardo, Florence Poy, F. Anthony Romero, Yong Tang, Jian Wang, Zhaowu Xu, Laura E. Zawadzke, Xiaoyu Zhu, Brian K. Albrecht, Steven R. Magnuson, Steve Bellon, and Andrea G. Cochran. Diving into the Water: Inducible Binding Conformations for BRD4, TAF1(2), BRD9, and CECR2 Bromodomains. *J. Med. Chem.*, 59(11):5391–5402, 2016. ISSN 15204804. doi:10.1021/acs.jmedchem.6b00264.

[24] Devashree N. Patil, Sushama A. Patil, Srinivas Sistla, and Jyoti P. Jadhav. Comparative biophysical characterization: A screening tool for acetylcholinesterase inhibitors. *PLoS One*, 14(5):1–28, 2019. ISSN 19326203. doi:10.1371/journal.pone.0215291.

[25] Yong Jia, Christopher M. Quinn, Anca Clabbers, Robert Talanian, Yajun Xu, Neil Wishart, and Hamish Allen. Comparative analysis of various in vitro COT kinase assay formats and their applications in inhibitor identification and characterization. *Anal. Biochem.*, 350(2):268–276, 2006. ISSN 10960309. doi:10.1016/j.ab.2005.11.010.

[26] Bradley A. Katz, Paul A. Sprengeler, Christine Luong, Erik Verner, Kyle Elrod, Matt Kirtley, James Janc, Jeffrey R. Spencer, J. Guy Breitenbucher, Hon Hui, Danny McGee, Darin Allen, Arnold Martelli, and Richard L. Mackman. Engineering inhibitors highly selective for the S1 sites of Ser190 trypsin-like serine protease drug targets. *Chem. Biol.*, 8(11):1107–1121, 2001. ISSN 10745521. doi:10.1016/S1074-5521(01)00084-9.

[27] Bradley A. Katz, Kyle Elrod, Erik Verner, Richard L. Mackman, Christine Luong, William D. Shrader, Martin Sendzik, Jeffrey R. Spencer, Paul A. Sprengeler, Aleks Kolesnikov, Vincent W.F. Tai, Hon C. Hui, J. Guy Breitenbucher, Darin Allen, and James W. Janc. Elaborate manifold of short hydrogen bond arrays mediating binding of active site-directed serine protease inhibitors. *J. Mol. Biol.*, 329(1):93–120, 2003. ISSN 00222836. doi:10.1016/S0022-2836(03)00399-1.

[28] Jiwang Chen, Yimei Wang, Ran Ye, Yongning Wua, and Wenshui Xia. Comparison of analytical methods to assay inhibitors of angiotensin I-converting enzyme. *Food Chem.*, 141(4):3329–3334, 2013. ISSN 18737072. doi:10.1016/j.foodchem.2013.06.048. URL `http://dx.doi.org/10.1016/j.foodchem.2013.06.048`.

[29] Hagit Achdout, Anthony Aimon, Elad Bar-David, Haim Barr, Amir Ben-Shmuel, James Bennett, Melissa L. Bobby, Juliane Brun, B. V.N.B.S. Sarma, Mark Calmiano, Anna Carbery, Emma Cattermole, John D. Chodera, Austin Clyde, Joseph E. Coffland, Galit Cohen, Jason Cole, Alessandro Contini, Lisa Cox, Milan Cvitkovic, Alex Dias, Alice Douangamath, Shirly Duberstein, Tim Dudgeon, Louise Dunnett, Peter K. Eastman, Noam Erez, Michael Fairhead, Daren Fearon, Oleg Fedorov, Matteo Ferla, Holly Foster, Richard Foster, Ronen Gabizon, Paul Gehrtz, Carina Gileadi, Charline Giroud, William G. Glass, Robert Glen, Itai Glinert, Marian Gorichko, Tyler Gorrie-Stone, Edward J. Griffen, Jag Heer, Michelle Hill, Sam Horrell, Matthew F.D. Hurley, Tomer Israely, Andrew Jajack, Eric Jnoff, Tobias John, Anastassia L. Kantsadi, Peter W. Kenny, John L. Kiappes, Lizbe Koekemoer, Boris Kovar, Tobias Krojer, Alpha Albert Lee, Bruce A. Lefker, Haim Levy, Nir London, Petra Lukacik, Hannah Bruce Macdonald, Beth MacLean, Tika R. Malla, Tatiana Matviiuk, Willam McCorkindale, Sharon Melamed, Oleg Michurin, Halina Mikolajek, Aaron Morris, Garrett M. Morris, Melody Jane Morwitzer, Demetri Moustakas, Jose Brandao Neto, Vladas Oleinikovas, Gijs J. Overheul, David Owen, Ruby Pai, Jin Pan, Nir Paran, Benjamin Perry, Maneesh Pingle, Jakir Pinjari, Boaz Politi, Ailsa Powell, Vladimir Psenak, Reut Puni, Victor L. Rangel, Rambabu N. Reddi, St Patrick Reid, Efrat Resnick, Matthew C. Robinson, Ralph P. Robinson, Dominic Rufa, Christopher Schofield, Aarif Shaikh, Jiye Shi, Khriesto Shurrush, Assa Sittner, Rachael Skyner, Adam Smalley, Mihaela D. Smilova, John Spencer, Claire Strain-Damerell, Vishwanath Swamy, Hadas Tamir, Rachael Tennant, Andrew Thompson, Warren Thompson, Susana Tomasio, Anthony Tumber, Ioannis Vakonakis, Ronald P. van Rij, Finny S. Varghese, Mariana Vaschetto, Einat B. Vitner, Vincent Voelz, Annette von Delft, Frank von Delft, Martin Walsh, Walter Ward, Charlie Weatherall, Shay Weiss, Conor Francis Wild, Matthew Wittmann, Nathan Wright, Yfat Yahalom-Ronen, Daniel Zaidmann, Hadeer Zidane, and Nicole Zitzmann. COVID moonshot: Open science discovery of SARS-CoV-2 main protease inhibitors by combining crowdsourcing, high-throughput experiments, computational simulations, and machine learning. *bioRxiv*, page 2020.10.29.339317, 2020. ISSN 26928205. URL `http://biorxiv.org/content/early/2020/10/30/2020.10.29.339317.abstract`.

[30] Anders Friberg, Dominico Vigil, Bin Zhao, R. Nathan Daniels, Jason P. Burke, Pedro M. Garcia-Barrantes, Demarco Camper, Brian A. Chauder, Taekyu Lee, Edward T. Olejniczak, and Stephen W. Fesik. Discovery of potent myeloid cell leukemia 1 (mcl-1) inhibitors using fragment-based methods and structure-based design. *Journal of Medicinal Chemistry*, 56:15–30, 1 2013. ISSN 00222623. doi:10.1021/jm301448p.

[31] Joseph W. Kaus, Edward Harder, Teng Lin, Robert Abel, J. Andrew McCammon, and Lingle Wang. How to deal with multiple binding poses in alchemical relative protein-ligand binding free energy calculations. *J. Chem. Theory Comput.*, 11(6):2670–2679, 2015. ISSN 15499626. doi:10.1021/acs.jctc.5b00214.

[32] David L Mobley, John D Chodera, and Ken A Dill. On the use of orientational restraints and symmetry corrections in alchemical free energy calculations. *J. Chem. Phys.*, 125(8), 2006. ISSN 00219606. doi:10.1063/1.2221683.

[33] Michael F.T. Koehler, Philippe Bergeron, Elizabeth M. Blackwood, Krista Bowman, Kevin R. Clark, Ron Firestein, James R. Kiefer, Klaus Maskos, Mark L. McCleland, Linda Orren, Laurent Salphati, Steve Schmidt, Elisabeth V. Schneider, Jiansheng Wu, and Maureen H. Beresini. Development of a potent, specific cdk8 kinase inhibitor which phenocopies cdk8/19 knockout cells. *ACS Medicinal Chemistry Letters*, 7:223–228, 1 2016. ISSN 19485875. doi:10.1021/acsmedchemlett.5b00278.

[34] Kai Schiemann, Aurélie Mallinger, Dirk Wienke, Christina Esdar, Oliver Poeschke, Michael Busch, Felix Rohdich, Suzanne A. Eccles, Richard Schneider, Florence I. Raynaud, Paul Czodrowski, Djordje Musil, Daniel Schwarz, Klaus Urbahns, and Julian Blagg. Discovery of potent and selective CDK8 inhibitors from an HSP90 pharmacophore. *Bioorganic Med. Chem. Lett.*, 26(5):1443–1451, 2016. ISSN 14643405. doi:10.1016/j.bmcl.2016.01.062. URL `http://dx.doi.org/10.1016/j.bmcl.2016.01.062`.

[35] Kai Schiemann, Dirk Finsinger, Frank Zenke, Christiane Amendt, Thorsten Knöchel, David Bruge, Hans Peter Buchstaller, Ulrich Emde, Wolfgang Stähle, and Soheila Anzali. The discovery and optimization of hexahydro-2H-pyrano[3,2-c]quinolines (HHPQs) as potent and selective inhibitors of the mitotic kinesin-5. *Bioorganic Med. Chem. Lett.*, 20(5):1491–1495, 2010. ISSN 0960894X. doi:10.1016/j.bmcl.2010.01.110. URL `http://dx.doi.org/10.1016/j.bmcl.2010.01.110`.

[36] Darryl David Dixon, Jonas Grina, John A. Josey, James P. Rizzi, Stephen T. Schlachter, Eli M. Wallace, Bin Wang, Paul Wehn, Rui Xu, and Hanbiao Yang. Aryl ethers and uses thereof, March 2018. URL `https://patents.google.com/patent/US9908845B2/en`. Patent No.: US 9,908,845 B2.

[37] Paul M. Wehn, James P. Rizzi, Darryl D. Dixon, Jonas A. Grina, Stephen T. Schlachter, Bin Wang, Rui Xu, Hanbiao Yang, Xinlin Du, Guangzhou Han, Keshi Wang, Zhaodan Cao, Tzuling Cheng, Robert M. Czerwinski, Barry S. Goggin, Heli Huang, Megan M. Halfmann, Melissa A. Maddie, Emily L. Morton, Sarah R. Olive, Huiling Tan, Shanhai Xie, Tai Wong, John A. Josey, and Eli M. Wallace. Design and Activity of Specific Hypoxia-Inducible Factor-2α (HIF-2α) Inhibitors for the Treatment of Clear Cell Renal Cell Carcinoma: Discovery of Clinical Candidate (S)-3-((2,2-Difluoro-1-hydroxy-7-(methylsulfonyl)-2,3-dihydro-1 H-inden-4-yl)oxy)-5-fluor. *J. Med. Chem.*, 61(21):9691–9721, 2018. ISSN 15204804. doi:10.1021/acs.jmedchem.8b01196.

[38] Jorge Garcia Fortanet, Christine Hiu Tung Chen, Ying Nan P. Chen, Zhouliang Chen, Zhan Deng, Brant Firestone, Peter Fekkes, Michelle Fodor, Pascal D. Fortin, Cary Fridrich, Denise Grunenfelder, Samuel Ho, Zhao B. Kang, Rajesh Karki, Mitsunori Kato, Nick Keen, Laura R. Labonte, Jay Larrow, Francois Lenoir, Gang Liu, Shumei Liu, Franco Lombardo, Dyuti Majumdar, Matthew J. Meyer, Mark Palermo, Lawrence Perez, Minying Pu, Timothy Ramsey, William R. Sellers, Michael D. Shultz, Travis Stams, Christopher Towler, Ping Wang, Sarah L. Williams, Ji Hu Zhang, and Matthew J. Lamarche. Allosteric Inhibition of SHP2: Identification of a Potent, Selective, and Orally Efficacious Phosphatase Inhibitor. *J. Med. Chem.*, 59(17):7773–7782, 2016. ISSN 15204804. doi:10.1021/acs.jmedchem.6b00680.

[39] Jeremy R. Greenwood, David Calkins, Arron P. Sullivan, and John C. Shelley. Towards the comprehensive, rapid, and accurate prediction of the favorable tautomeric states of drug-like molecules in aqueous solution. *J. Comput. Aided. Mol. Des.*, 24:591–604, 2010. ISSN 0920654X. doi:10.1007/s10822-010-9349-1.

[40] Dean M. Philipp, Mark A. Watson, Haoyu S. Yu, Thomas B. Steinbrecher, and Art D. Bochevarov. Quantum chemical pK a prediction for complex organic molecules. *Int. J. Quantum Chem.*, 118(12):1–8, 2018. ISSN 1097461X. doi:10.1002/qua.25561.

[41] Hans Peter Buchstaller, Uwe Anlauf, Dieter Dorsch, Daniel Kuhn, Martin Lehmann, Birgitta Leuthner, Djordje Musil, Daniela Radtki, Claudio Ritzert, Felix Rohdich, Richard Schneider, and Christina Esdar. Discovery and Optimization of 2-Arylquinazolin-4-ones into a Potent and Selective Tankyrase Inhibitor Modulating Wnt Pathway Activity. *J. Med. Chem.*, 62(17):7897–7909, 2019. ISSN 15204804. doi:10.1021/acs.jmedchem.9b00656.

[42] César De Oliveira, Haoyu S. Yu, Wei Chen, Robert Abel, and Lingle Wang. Rigorous Free Energy Perturbation Approach to Estimating Relative Binding Affinities between Ligands with Multiple Protonation and Tautomeric States. *J. Chem. Theory Comput.*, 15(1):424–435, 2019. ISSN 15499626. doi:10.1021/acs.jctc.8b00826.

[43] Thomas B. Steinbrecher, Markus Dahlgren, Daniel Cappel, Teng Lin, Lingle Wang, Goran Krilov, Robert Abel, Richard Friesner, and Woody Sherman. Accurate Binding Free Energy Predictions in Fragment Optimization. *J. Chem. Inf. Model.*, 55(11):2411–2420, 2015. ISSN 15205142. doi:10.1021/acs.jcim.5b00538.

[44] John J. Barker, Oliver Barker, Roberto Boggio, Viddhata Chauhan, Robert K.Y. Cheng, Vincent Corden, Stephen M. Courtney, Neil Edwards, Virginie M. Falque, Fulvia Fusar, Mihaly Gardiner, Estelle M.N. Hamelin, Thomas Hesterkamp, Osamu Ichihara, Richard S. Jones, Owen Mather, Ciro Mercurio, Saverio Minucci, Christian A.G.N. Montalbetti, Annett Müller, Deepti Patel, Banu G. Phillips, Mario Varasi, Mark Whittaker, Dirk Winkler, and Christopher J. Yarnold. Fragment-based identification of Hsp90 inhibitors. *ChemMedChem*, 4(6):963–966, 2009. ISSN 18607179. doi:10.1002/cmdc.200900011.

[45] Stephen Antonysamy, Gavin Hirst, Frances Park, Paul Sprengeler, Frank Stappenbeck, Ruo Steensma, Mark Wilson, and Melissa Wong. Fragment-based discovery of JAK-2 inhibitors. *Bioorganic Med. Chem. Lett.*, 19 (1):279–282, 2009. ISSN 0960894X. doi:10.1016/j.bmcl.2008.08.064. URL `http://dx.doi.org/10.1016/j.bmcl.2008.08.064`.

[46] Francesco Deflorian, Laura Pérez-Benito, Eelke B. Lenselink, Miles Congreve, Herman Van Vlijmen, Jonathan S. Mason, Chris de Graaf, and Gary Tresadern. Accurate Prediction of GPCR Ligand Binding Affinity with Free Energy Perturbation. *J. Chem. Inf. Model.*, 2020. ISSN 1549-9596. doi:10.1021/acs.jcim.0c00449.

[47] Callum J Dickson, Viktor Hornak, and Jose S Duca. Relative Binding Free-Energy Calculations at Lipid-Exposed Sites: Deciphering Hot Spots. *J. Chem. Inf. Model.*, 61(12):5923–5930, 2021. ISSN 15205142. doi:10.1021/acs.jcim.1c01147.

[48] Zhan Guo Gao, Qiaoling Jiang, Kenneth A. Jacobson, and Adriaan P. Ijzerman. Site-directed mutagenesis studies of human A(2A) adenosine receptorsInvolvement of glu13 and his278 in ligand binding and sodium modulation. *Biochem. Pharmacol.*, 60(5):661–668, 2000. ISSN 00062952. doi:10.1016/S0006-2952(00)00357-9.

[49] Miles Congreve, Stephen P. Andrews, Andrew S. Doré, Kaspar Hollenstein, Edward Hurrell, Christopher J. Langmead, Jonathan S. Mason, Irene W. Ng, Benjamin Tehan, Andrei Zhukov, Malcolm Weir, and Fiona H. Marshall. Discovery of 1,2,4-triazine derivatives as adenosine A 2A antagonists using structure based drug design. *J. Med. Chem.*, 55(5):1898–1903, 2012. ISSN 00222623. doi:10.1021/jm201376w.

[50] Dandan Zhang, Zhan Guo Gao, Kaihua Zhang, Evgeny Kiselev, Steven Crane, Jiang Wang, Silvia Paoletta, Cuiying Yi, Limin Ma, Wenru Zhang, Gye Won Han, Hong Liu, Vadim Cherezov, Vsevolod Katritch, Hualiang Jiang, Raymond C. Stevens, Kenneth A. Jacobson, Qiang Zhao, and Beili Wu. Two disparate ligand-binding sites in the human P2Y1 receptor. *Nature*, 520(7547):317–321, 2015. ISSN 14764687. doi:10.1038/nature14287.

[51] Hannguang Chao, Huji Turdi, Timothy F. Herpin, Jacques Y. Roberge, Yalei Liu, Dora M. Schnur, Michael A. Poss, Robert Rehfuss, Ji Hua, Qimin Wu, Laura A. Price, Lynn M. Abell, William A. Schumacher, Jeffrey S. Bostwick, Thomas E. Steinbacher, Anne B. Stewart, Martin L. Ogletree, Christine S. Huang, Ming Chang, Angela M. Cacace, Maredith J. Arcuri, Deborah Celani, Ruth R. Wexler, and R. Michael Lawrence. Discovery of 2-(phenoxypyridine)-3-phenylureas as small molecule P2Y 1 antagonists. *J. Med. Chem.*, 56(4):1704–1714, 2013. ISSN 15204804. doi:10.1021/jm301708u.

[52] Mikhail A. Lomize, Andrei L. Lomize, Irina D. Pogozheva, and Henry I. Mosberg. OPM: Orientations of proteins in membranes database. *Bioinformatics*, 22(5):623–625, 2006. ISSN 13674803. doi:10.1093/bioinformatics/btk023.

[53] Myriam Ciordia, Laura Pérez-Benito, Francisca Delgado, Andrés A. Trabanco, and Gary Tresadern. Application of Free Energy Perturbation for the Design of BACE1 Inhibitors. *J. Chem. Inf. Model.*, 56(9):1856–1871, 2016. ISSN 15205142. doi:10.1021/acs.jcim.6b00220.

[54] Henrik Keränen, Laura Pérez-Benito, Myriam Ciordia, Francisca Delgado, Thomas B. Steinbrecher, Daniel Oehlrich, Herman W.T. Van Vlijmen, Andrés A. Trabanco, and Gary Tresadern. Acylguanidine Beta Secretase 1 Inhibitors: A Combined Experimental and Free Energy Perturbation Study. *J. Chem. Theory Comput.*, 13(3): 1439–1453, 2017. ISSN 15499626. doi:10.1021/acs.jctc.6b01141.

[55] Hideaki Shimizu, Asako Tosaki, Kumi Kaneko, Tamao Hisano, Takashi Sakurai, and Nobuyuki Nukina. Crystal Structure of an Active Form of BACE1, an Enzyme Responsible for Amyloid β Protein Production. *Mol. Cell. Biol.*, 28(11):3663–3671, 2008. ISSN 0270-7306. doi:10.1128/mcb.02185-07.

[56] Vincent Wagner, Linda Jantz, Hans Briem, Kai Sommer, Matthias Rarey, and Clara D. Christ. Computational Macrocyclization: From de novo Macrocycle Generation to Binding Affinity Estimation. *ChemMedChem*, 12(22): 1866–1872, 2017. ISSN 18607187. doi:10.1002/cmdc.201700478.

[57] Haoyu S. Yu, Yuqing Deng, Yujie Wu, Dan Sindhikara, Amy R. Rask, Takayuki Kimura, Robert Abel, and Lingle Wang. Accurate and Reliable Prediction of the Binding Affinities of Macrocycles to Their Protein Targets. *J. Chem. Theory Comput.*, 13(12):6290–6300, 2017. ISSN 15499626. doi:10.1021/acs.jctc.7b00885.

[58] E. Scott Priestley, Daniel L. Cheney, Indawati DeLucca, Anzhi Wei, Joseph M. Luettgen, Alan R. Rendina, Pancras C. Wong, and Ruth R. Wexler. Structure-Based Design of Macrocyclic Coagulation Factor VIIa Inhibitors. *J. Med. Chem.*, 58(15):6225–6236, 2015. ISSN 15204804. doi:10.1021/acs.jmedchem.5b00788.

[59] Ian M. Bell, Steven N. Gallicchio, Marc Abrams, Lorena S. Beese, Douglas C. Beshore, Hema Bhimnathwala, Michael J. Bogusky, Carolyn A. Buser, J. Christopher Culberson, Joseph Davide, Michelle Ellis-Hutchings, Christine Fernandes, Jackson B. Gibbs, Samuel L. Graham, Kelly A. Hamilton, George D. Hartman, David C. Heimbrook, Carl F. Homnick, Hans E. Huber, Joel R. Huff, Kelem Kassahun, Kenneth S. Koblan, Nancy E. Kohl, Robert B. Lobell, Joseph J. Lynch, Ronald Robinson, A. David Rodrigues, Jeffrey S. Taylor, Eileen S. Walsh, Theresa M. Williams, and C. Blair Zartmant. 3-Aminopyrrolidinone farnesyltransferase inhibitors: Design of macrocyclic compounds with improved pharmacokinetics and excellent cell potency. *J. Med. Chem.*, 45(12): 2388–2409, 2002. ISSN 00222623. doi:10.1021/jm010531d.

[60] Daniel Cappel, Steven Jerome, Gerhard Hessler, and Hans Matter. Impact of Different Automated Binding Pose Generation Approaches on Relative Binding Free Energy Simulations. *J. Chem. Inf. Model.*, 60(3):1432–1444, 2020. ISSN 15205142. doi:10.1021/acs.jcim.9b01118.

[61] Henning Steinhagen, Bodo Scheiper, Hans Matter, Hans Ulrich Stilz, and Gary Mccort. Cyclic indole-3-carboxamides, their preparation and their use as pharmaceuticals, April 2009. URL `https://patents.google.com/patent/WO2009095163A3`. Patent No.: WO 2009/095163 A3.

[62] Franz Vonnussbaum, Volkhart M.J. Li, Swen Allerheiligen, Sonja Anlauf, Lars Bärfacker, Martin Bechem, Martina Delbeck, Mary F. Fitzgerald, Michael Gerisch, Heike Gielen-Haertwig, Helmut Haning, Dagmar Karthaus, Dieter Lang, Klemens Lustig, Daniel Meibom, Joachim Mittendorf, Ulrich Rosentreter, Martina Schäfer, Stefan Schäfer, Jens Schamberger, Leila A. Telan, and Adrian Tersteegen. Freezing the Bioactive Conformation to Boost Potency: The Identification of BAY85-8501, a Selective and Potent Inhibitor of Human Neutrophil Elastase for Pulmonary Diseases. *ChemMedChem*, 10(7):1163–1173, 2015. ISSN 18607187. doi:10.1002/cmdc.201500131.

[63] Lingle Wang, Yuqing Deng, Yujie Wu, Byungchan Kim, David N Lebard, Dan Wandschneider, Mike Beachy, Richard A Friesner, and Robert Abel. Accurate Modeling of Scaffold Hopping Transformations in Drug Discovery. *J. Chem. Theory Comput.*, 13:42–54, 2017. doi:10.1021/acs.jctc.6b00991.

[64] Atsushi Suda, Hiroshi Koyano, Tadakatsu Hayase, Kihito Hada, Ken Ichi Kawasaki, Susumu Komiyama, Kiyoshi Hasegawa, Takaaki A. Fukami, Shigeo Sato, Takaaki Miura, Naomi Ono, Toshikazu Yamazaki, Ryoichi Saitoh, Nobuo Shimma, Yasuhiko Shiratori, and Takuo Tsukuda. Design and synthesis of novel macrocyclic 2-amino-6-arylpyrimidine Hsp90 inhibitors. *Bioorganic Med. Chem. Lett.*, 22(2):1136–1141, 2012. ISSN 0960894X. doi:10.1016/j.bmcl.2011.11.100. URL `http://dx.doi.org/10.1016/j.bmcl.2011.11.100`.

[65] Gregory A. Ross, Ellery Russell, Yuqing Deng, Chao Lu, Edward D. Harder, Robert Abel, and Lingle Wang. Enhancing Water Sampling in Free Energy Calculations with Grand Canonical Monte Carlo. *J. Chem. Theory Comput.*, 2020. ISSN 1549-9618. doi:10.1021/acs.jctc.0c00660.

[66] Chao Lu, Chuanjie Wu, Delaram Ghoreishi, Wei Chen, Lingle Wang, Wolfgang Damm, Gregory A. Ross, Markus K. Dahlgren, Ellery Russell, Christopher D. Von Bargen, Robert Abel, Richard A. Friesner, and Edward D. Harder. OPLS4: Improving Force Field Accuracy on Challenging Regimes of Chemical Space. *J. Chem. Theory Comput.*, 2021. ISSN 1549-9618. doi:10.1021/acs.jctc.1c00302.

[67] Wei Chen, Yuqing Deng, Ellery Russell, Yujie Wu, Robert Abel, and Lingle Wang. Accurate Calculation of Relative Binding Free Energies between Ligands with Different Net Charges. *J. Chem. Theory Comput.*, 14(12):6346–6358, 2018. ISSN 15499626. doi:10.1021/acs.jctc.8b00825.

[68] Arjun Saha, Amy Y. Shih, Taraneh Mirzadegan, and Mark Seierstad. Predicting the Binding of Fatty Acid Amide Hydrolase Inhibitors by Free Energy Perturbation. *J. Chem. Theory Comput.*, 14(11):5815–5822, 2018. ISSN 15499626. doi:10.1021/acs.jctc.8b00672.

[69] Francesco Manzoni and Ulf Ryde. Assessing the stability of free-energy perturbation calculations by performing variations in the method. *J. Comput. Aided. Mol. Des.*, 32(4):529–536, 2018. ISSN 15734951. doi:10.1007/s10822-018-0110-5. URL `http://dx.doi.org/10.1007/s10822-018-0110-5`.

[70] Vytautas Gapsys, Laura Pérez-Benito, Matteo Aldeghi, Daniel Seeliger, Herman Van Vlijmen, Gary Tresadern, and Bert L. De Groot. Large scale relative protein ligand binding affinities using non-equilibrium alchemy. *Chem. Sci.*, 11(4):1140–1152, 2020. ISSN 20416539. doi:10.1039/c9sc03754c.

[71] Christopher R. Smith, Douglas R. Dougan, Mallareddy Komandla, Toufike Kanouni, Beverly Knight, J. David Lawson, Mark Sabat, Ewan R. Taylor, Phong Vu, and Corey Wyrick. Fragment-Based Discovery of a Small Molecule Inhibitor of Bruton's Tyrosine Kinase. *J. Med. Chem.*, 58(14):5437–5444, 2015. ISSN 15204804. doi:10.1021/acs.jmedchem.5b00734.

[72] Jenny K Ekegren, Nina Ginman, Åsa Johansson, Hans Wallberg, Mats Larhed, Bertil Samuelsson, Torsten Unge, and Anders Hallberg. Microwave-Accelerated Synthesis of P1'-Extended HIV-1 Protease Inhibitors Encompassing a Tertiary Alcohol in the Transition-State Mimicking Scaffold. *J. Med. Chem.*, 49:1828–1832, 2006. doi:10.1021/jm051239z CCC:.

[73] Lingle Wang, Yujie Wu, Yuqing Deng, Byungchan Kim, Levi Pierce, Goran Krilov, Dmitry Lupyan, Shaughnessy Robinson, Markus K Dahlgren, Jeremy Greenwood, Donna L Romero, Craig Masse, Jennifer L Knight, Thomas Steinbrecher, Thijs Beuming, Wolfgang Damm, Ed Harder, Woody Sherman, Mark Brewer, Ron Wester, Mark Murcko, Leah Frye, Ramy Farid, Teng Lin, David L Mobley, William L Jorgensen, Bruce J Berne, Richard A Friesner, and Robert Abel. Accurate and Reliable Prediction of Relative Ligand Binding Potency in Prospective Drug Discovery by Way of a Modern Free-Energy Calculation Protocol and Force Field. *J. Am. Chem. Soc.*, 137 (7):2695–2703, feb 2015. doi:10.1021/ja512751q. URL `http://dx.doi.org/10.1021/ja512751q`.

[74] Katarina Roos, Chuanjie Wu, Wolfgang Damm, Mark Reboul, James M. Stevenson, Chao Lu, Markus K. Dahlgren, Sayan Mondal, Wei Chen, Lingle Wang, Robert Abel, Richard A. Friesner, and Edward D. Harder. OPLS3e: Extending Force Field Coverage for Drug-Like Small Molecules. *J. Chem. Theory Comput.*, 15(3):1863–1874, 2019. ISSN 15499626. doi:10.1021/acs.jctc.8b01026.

[75] Kenji Yoshikawa, Aki Yokomizo, Hiroyuki Naito, Noriyasu Haginoya, Shozo Kobayashi, Toshiharu Yoshino, Tsutomu Nagata, Akiyoshi Mochizuki, Ken Osanai, Kengo Watanabe, Hideyuki Kanno, and Toshiharu Ohta. Design, synthesis, and SAR of cis-1,2-diaminocyclohexane derivatives as potent factor Xa inhibitors. Part I: Exploration of 5-6 fused rings as alternative S1 moieties. *Bioorganic Med. Chem.*, 17(24):8206–8220, 2009. ISSN 09680896. doi:10.1016/j.bmc.2009.10.023. URL `http://dx.doi.org/10.1016/j.bmc.2009.10.023`.

[76] Kenji Yoshikawa, Shozo Kobayashi, Yumi Nakamoto, Noriyasu Haginoya, Satoshi Komoriya, Toshiharu Yoshino, Tsutomu Nagata, Akiyoshi Mochizuki, Kengo Watanabe, Makoto Suzuki, Hideyuki Kanno, and Toshiharu Ohta. Design, synthesis, and SAR of cis-1,2-diaminocyclohexane derivatives as potent factor Xa inhibitors. Part II: Exploration of 6-6 fused rings as alternative S1 moieties. *Bioorganic Med. Chem.*, 17(24):8221–8233, 2009. ISSN 09680896. doi:10.1016/j.bmc.2009.10.024. URL `http://dx.doi.org/10.1016/j.bmc.2009.10.024`.
